# A two-step adaptive walk rewires nutrient transport in a challenging edaphic environment

**DOI:** 10.1126/sciadv.abm9385

**Published:** 2022-05-18

**Authors:** Emmanuel Tergemina, Ahmed F. Elfarargi, Paulina Flis, Andrea Fulgione, Mehmet Göktay, Célia Neto, Marleen Scholle, Pádraic J. Flood, Sophie-Asako Xerri, Johan Zicola, Nina Döring, Herculano Dinis, Ute Krämer, David E. Salt, Angela M. Hancock

**Affiliations:** 1 Department of Plant Developmental Biology, Max Planck Institute for Plant Breeding Research, 50829 Cologne, Germany.; 2 Future Food Beacon of Excellence and the School of Biosciences, University of Nottingham, Sutton Bonington Campus, Nr Loughborough, LE12 5RD Nottingham, UK.; 3 Faculty of Biology and Biotechnology, Ruhr University Bochum, 44801 Bochum, Germany.; 4 Parque Natural do Fogo, Direção Nacional do Ambiente, 115 Chã d’Areia, Praia, Santiago, Cabo Verde, Africa.; 5 Associação Projecto Vitó, 8234, Xaguate, Cidade de São Filipe, Fogo, Cabo Verde, Africa.

## Abstract

Most well-characterized cases of adaptation involve single genetic loci. Theory suggests that multilocus adaptive walks should be common, but these are challenging to identify in natural populations. Here, we combine trait mapping with population genetic modeling to show that a two-step process rewired nutrient homeostasis in a population of *Arabidopsis* as it colonized the base of an active stratovolcano characterized by extremely low soil manganese (Mn). First, a variant that disrupted the primary iron (Fe) uptake transporter gene (*IRT1*) swept quickly to fixation in a hard selective sweep, increasing Mn but limiting Fe in the leaves. Second, multiple independent tandem duplications occurred at *NRAMP1* and together rose to near fixation in the island population, compensating the loss of IRT1 by improving Fe homeostasis. This study provides a clear case of a multilocus adaptive walk and reveals how genetic variants reshaped a phenotype and spread over space and time.

## Teaser

A hard sweep at *IRT1* followed by a soft sweep at *NRAMP1* underlies adaptation.

## INTRODUCTION

Determining the genetic mechanisms underlying adaptation to challenging environments is a central goal in biology; it also has practical applications for a broad range of critical issues in agriculture, conservation, and medicine. Potential uses include developing sustainable crops tailored to local environments, managing vulnerable populations and species, and mitigating emerging risks from pathogens.

While several cases of single-gene effects on putatively adaptive phenotypes have been described, e.g., (*1*–*5*), theory and data suggest that most traits are polygenic. Thus, after colonization of a novel environment, adaptation is likely to involve changes at multiple loci, producing multistep walks toward higher fitness (*6*–*11*). However, we lack concrete examples of multilocus processes in wild populations. As a result, there are many open questions. For example, are multilocus adaptive processes likely to involve proteins that interact at the molecular level or those that have distinct cellular functions? To what extent does the first evolutionary step limit subsequent steps? Do epistasis and pleiotropy limit evolutionary paths? Does the mode of adaptation fit a “hard selective sweep” model (*12*), where a single mutation rises rapidly to fixation in the population, or does it occur through “soft selective sweeps” (*13*, *14*), where multiple mutations rise in frequency as a group? To address these questions in the context of multistep adaptive processes, we need detailed information about specific cases, including the precise functional variants involved, their physiological consequences, and reconstructions of their spread over time and space.

**Fig. 1.**
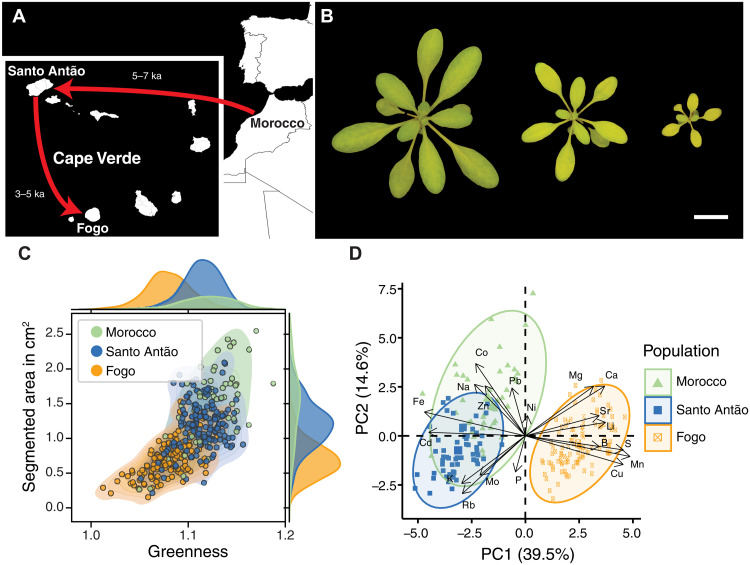
Fogo plants have a distinct leaf ionome, characterized by Mn enrichment and Fe deficiency. (**A**) Schematic of the colonization of Fogo from Santo Antão. (**B**) Representative lines from Fogo showing high variation in size and greenness on standard potting mix. Scale bar, 1 cm. (**C**) Bivariate distribution of greenness (green/red) and segmented area (cm^2^) by population [Fogo (orange), Santo Antão (blue), and Morocco (green)]. Phenotype distributions are shown on the axes. (**D**) Fogo samples separate from Moroccan and Santo Antão samples on the first principal component of a matrix of leaf elemental concentrations, color-coded as in (C). In (C) and (D), each dot corresponds to the median value across replicates.

We capitalize on a case where *Arabidopsis* recently colonized an active stratovolcano with a harsh edaphic environment to examine the genetic steps that led to adaptation. *Arabidopsis* colonized Fogo ~3 to 5 ka from Santo Antão, an island ~250 km to the north (Fig. 1A) (*15*). Fogo and Santo Antão are both volcanic islands in the Cape Verde archipelago, but the two islands have different pedogenic histories. While Fogo is an active stratovolcano, which experienced several eruptions over the past few thousand years, including the most recent in 2015, the last eruption on Santo Antão was much more ancient (~90 ka) (*16*). Today, the two island populations are genetically distinct from one another and from the continental populations due to severe colonization bottlenecks, which eliminated 99.4% of all shared variation, resulting in a phylogenetic speciation event (*15*).

## RESULTS

### Fogo plants have a distinct leaf ionome compared to Santo Antão and Morocco

When we propagated Fogo accessions in standard growth conditions, we found that they exhibited varying degrees of chlorosis, a phenotype we had not observed in native habitats (Fig. 1B). Quantitative image analysis showed that Fogo accessions were less green and smaller than their closest relatives in Santo Antão [Kruskal-Wallis (KW), greenness: *P* = 3.72 × 10^−30^, segmented area: *P* = 3.98 × 10^−29^] and Morocco (KW, greenness: *P* = 1.49 × 10^−18^, segmented area: *P* = 1.15 × 10^−18^) (Fig. 1C), and both traits were strongly correlated with relative chlorophyll content (greenness: Pearson’s *R*^2^ = 0.56, *n* = 31, *P* = 1.38 × 10^−6^; segmented area: Pearson’s *R*^2^ = 0.64, *n* = 31, *P* = 6.72 × 10^−8^) (fig. S1). In the most affected accessions, photosynthetic efficiency (Phi2) was severely reduced (fig. S2). Because chlorosis is often caused by mineral nutrient imbalance, we investigated the leaf ionome to determine whether the Fogo population showed differences relative to Santo Antão and Morocco. The concentrations of several mineral nutrients and trace elements were markedly shifted in Fogo accessions (fig. S3), resulting in a clear separation in multivariate clustering [principal components analysis (PCA)] (Fig. 1D). Mn and Fe were the major contributors to this separation, with loadings of 11.53 and 10.73% on PC1, which explained 39.5% of the variation (fig. S4 and table S1). Mn levels were ~4-fold higher in leaves of Fogo plants compared to those from Santo Antão and Morocco [Fogo mean = 501.27 μg g^−1^ dry weight (DW) ± 5.62 standard error (SE); Santo Antão mean = 133.37 μg g^−1^ DW ± 1.47 SE; Morocco mean = 133.81 μg g^−1^ DW ± 2.20 SE]. Consistent with the observed chlorosis, Fe levels were significantly lower in Fogo leaves compared to plants from Santo Antão and Morocco (Fogo mean = 52.73 μg g^−1^ DW ± 0.45 SE; Santo Antão mean = 79.90 μg g^−1^ DW ± 0.84 SE; Morocco mean = 79.11 μg g^−1^ DW ± 0.79 SE) (fig. S3 and table S2).

The parent material from which volcanic soils develop results from magmatism and often has chemical characteristics that limit plant growth. These include high phosphorous retention and micronutrient insufficiencies (*17*). To characterize the nutrient composition of the native soil, we collected topsoil samples from *Arabidopsis* stands across Fogo and Santo Antão. Topsoil analyses indicated that, in comparison to Santo Antão, Fogo soils are more alkaline (Fogo pH mean = 7.7; Santo Antão pH mean = 7) and impoverished in several mineral nutrients (tables S3 and S4). Among the 22 water-extractable elements tested, Fogo and Santo Antão soils differed most significantly in Co, Fe, K/Rb, Ni, Mn, and P (fig. S5 and table S4). To more directly estimate the availability of elements in the native soil, we characterized leaf ionomes of Santo Antão (S1-1) and Fogo (F13-8) accessions grown on Fogo and Santo Antão soils in comparison to standard potting mix. Mn levels were very low for plants grown on Fogo soil (S1-1 mean = 44.18 μg g^−1^ DW ± 9.82 SE, F13-8 mean = 72.30 μg g^−1^ DW ± 3.73 SE) compared to Santo Antão soil (S1-1 mean = 101.27 μg g^−1^ DW ± 13.93 SE, F13-8 mean= 114.98 μg g^−1^ DW ± 8.63 SE) and standard potting mix (S1-1 mean = 155.34 μg g^−1^ DW ± 7.64 SE, F13-8 mean = 208.10 μg g^−1^ DW ± 6.11 SE) (fig. S6) and were near the critical Mn deficiency concentration observed in plants (20 μg g^−1^ DW) (*18*). Our results show that Fogo *Arabidopsis* grows on soils with low bioavailable Mn. Further, the enrichment of Mn in the leaves of Fogo plants suggests that the Fogo plants have adapted to enhance Mn transport into leaf tissues under Mn limitation imposed by the Fogo soil.

### Mapping reveals a loss-of-function variant of *IRT1* and a segmental tandem triplication at *NRAMP1*

**Fig. 2.**
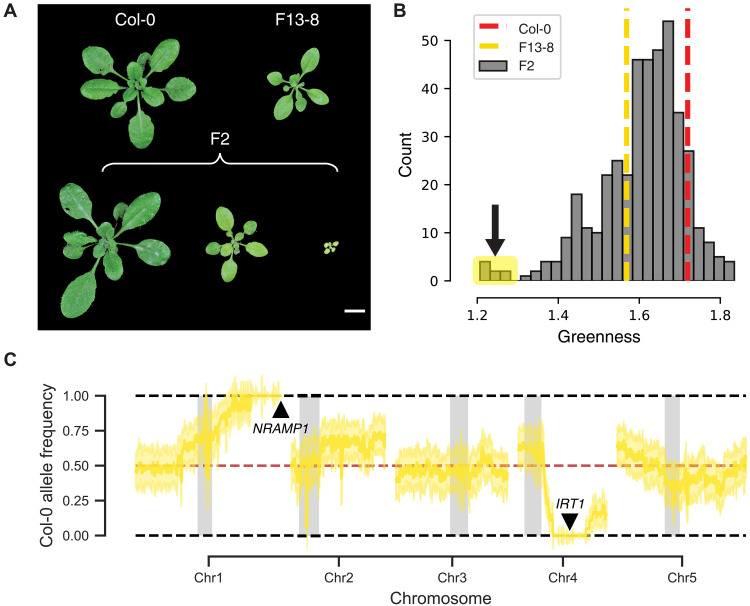
Mapping reveals a loss-of-function variant of *IRT1* and a segmental tandem triplication at *NRAMP1*. (**A**) F2 progeny derived from a Col-0 × F13-8 cross vary in size and greenness on standard potting mix. Scale bar, 1 cm. (**B**) Quantitative measure of greenness in 454 F2 progeny reveals a subset of extremely chlorotic segregants. Red and yellow dashed lines represent the averages for replicates of Col-0 and F13-8, respectively. The arrow and yellow rectangle indicate the subpopulation of highly chlorotic plants used for BSA in (C). (**C**) Chlorotic individuals are fixed for the reference (Col-0) *NRAMP1* allele and the Fogo (F13-8) *IRT1* allele. Median allele frequency using 200-kb sliding windows with a step size of 5 kb (yellow line) and corresponding SD (yellow shade). Red dashed line indicates 0.5 allele frequency. Gray rectangles specify centromeric regions.

To investigate the genetic basis of the Fogo chlorosis/small-size phenotype in standard potting mix, we examined segregation of the trait in an F2 population created from a cross between a Fogo accession (F13-8) and the *Arabidopsis thaliana* reference accession (Col-0). We found that roughly 1 of 16 individuals were highly chlorotic [*X*^2^ (1, *n* = 454) = 0.41, *P* = 0.52] (Fig. 2, A and B), suggesting that a two-locus model could explain the chlorotic phenotype. We conducted bulk segregant analysis (BSA) by sequencing five pools of Col-0 × F13-8 F2s with varying degrees of chlorosis (fig. S7). Sequencing of the most chlorotic pool revealed large deviations from the expected frequencies at two genomic regions. Severe chlorosis was linked to the combination of the Col-0 region at the end of chromosome 1 and the F13-8 region on chromosome 4 (Fig. 2C and fig. S7). We next examined the BSA peaks for potential causative variants. No single-nucleotide polymorphism (SNP) variant stood out as a strong functional candidate in the chromosome 1 region, but in the chromosome 4 region, we identified an SNP in the first exon of *IRON REGULATED TRANSPORTER 1* (*IRT1*), leading to a premature stop codon at position 130 (*IRT1* G130X) (fig. S8 and tables S5 and S6). *IRT1* encodes a divalent cation transporter that primarily contributes to Fe uptake at the root surface in *A. thaliana* and therefore represents an excellent candidate. Indeed, *irt1* mutants exhibit severe chlorosis (*19*–*21*), reminiscent of the phenotype we observed in the most chlorotic pool of Col-0 × F13-8 segregants, supporting a causative role of *IRT1* 130X in the chlorotic phenotype.

Next, we more closely examined the BSA peak in the chromosome 1 region and found a ~15-kb region surrounding *NATURAL RESISTANCE-ASSOCIATED MACROPHAGE PROTEIN 1* (*NRAMP1*), where read coverage was ~3-fold higher than the surrounding genomic region, suggesting gene copy number variation (CNV) (fig. S9 and table S7). To confirm this, we generated a whole-genome de novo assembly with Nanopore sequencing technology for F13-8 and identified a segmental tandem triplication at the *NRAMP1* region (fig. S9B). We asked whether the CNV was associated with increased *NRAMP1* expression in root tissue and found (~2.2-fold) higher *NRAMP1* mRNA levels in F13-8 compared to Col-0 (*t* test, *P* = 3.0 × 10^−3^) (fig. S9C). *NRAMP1* encodes a metal transporter crucial for growth in Mn-deficient conditions that cooperates with IRT1 for Fe transport (*22*–*24*). To determine whether the NRAMP1 protein in Fogo may show a different substrate spectrum compared to the Santo Antão outgroup population, we compared NRAMP1 protein sequences in Fogo and Santo Antão plants to the Col-0 reference. We found no segregating amino acid variation at NRAMP1 within Cape Verde and only a single amino acid change between the Cape Verdean accessions and Col-0 (L202I), which is unlikely to change its transport activity (fig. S10). Overall, our results show that the *NRAMP1* CNV is a strong candidate to explain the chromosome 1 peak.

We further validated that these two loci could largely explain the observed phenotypic variation in the Col-0 × F13-8 F2 population by genotyping each F2 individual at *IRT1* and *NRAMP1* [analysis of variance (ANOVA), *F* = 23.5031, *P* < 2.2 × 10^−16^] (fig. S11 and table S8). Further, the *IRT1* and *NRAMP1* mutations exhibited epistasis such that the effect of the *NRAMP1* CNV was only apparent in *IRT1* 130X homozygotes (table S9). Together, the BSA results provided compelling explanations for the Fe deficiency and the Mn enrichment observed in the leaves of the Fogo plants.

### Loss of IRT1 exerts strong pleiotropic effects on nutrient transport in the Fogo natural population, increasing Mn in the leaves but inducing Fe deficiency

Mapping in the recombinant population provided functional loci in a single Fogo individual (F13-8). We next examined the frequencies and effects of these loci in the broader context of the population and species. We found that *IRT1* 130X is fixed in Fogo and absent in the closest outgroup populations (Santo Antão and Morocco). Moreover, we found no evidence for any other *IRT1* loss-of-function variant in worldwide polymorphism data (table S10) (*25*–*28*). Given that plant growth in *irt1* mutants is severely altered (*19*–*21*), the fixation of the *IRT1* truncation in Fogo was unexpected and led us to investigate its physiological effect as it occurred in Fogo.

**Fig. 3.**
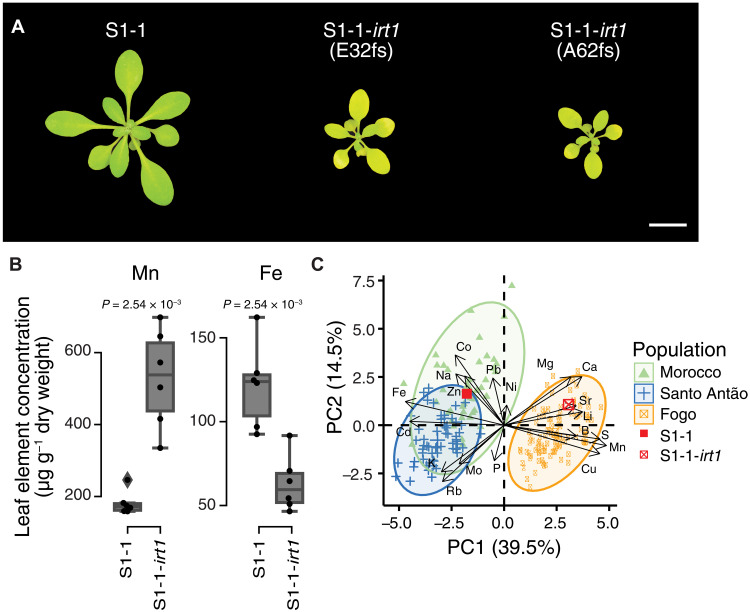
The loss of IRT1 exerts strong pleiotropic effects on nutrient transport in Fogo, increasing Mn in the leaves but inducing Fe deficiency. CRISPR to disrupt *IRT1* function in a Santo Antão individual (S1-1) exhibits (**A**) chlorosis and (**B**) increased Mn and reduced Fe in leaves, consistent with a strong role for the loss of IRT1 in overall pattern of differences observed between Santo Antão and Fogo populations. In (B), each dot represents one replicate per genotype. *P* = *P* value for MMW test. (**C**) CRISPR-induced loss of IRT1 in a Santo Antão individual (S1-1) results in a shift into the Fogo cloud. Each dot represents the median value across replicates. In (B) and (C), S1-1-*irt1* corresponds to S1-1-*irt1*(E32fs).

To achieve this, we used the CRISPR-Cas9 system to generate a knockout allele of *IRT1* in S1-1, an accession from Santo Antão and the closest outgroup, which carries both the ancestral *IRT1* (functional) and *NRAMP1* (single-copy) haplotype (Fig. 3A and fig. S12). On standard potting mix, S1-1-*irt1* plants were chlorotic, but the severity of this phenotype was milder than the Col-0-*irt1* mutant, suggesting that other loci contribute to the difference between Col-0-*irt1* and S1-1-*irt1* (figs. S13 and S14). This genetic background effect may not be unexpected given the high divergence between the Cape Verde Islands (CVI) accessions and Eurasians, including the Col-0 accession. While outside of the scope of this study, further examination of variation in the impact of IRT1 loss in diverse *A. thaliana* accessions would be warranted. We found that supplementing S1-1-*irt1* with Fe reduces chlorosis in standard potting soil (fig. S14). As expected, loss of IRT1 in a Santo Antão background caused a significant reduction in leaf Fe [Mann-Whitney-Wilcoxon (MMW) test, *P* = 4.06 × 10^−3^] (Fig. 3B and figs. S13, S15, and S16), but it also caused an unexpected ~3-fold increase in leaf levels of Mn (MMW test, *P* = 4.06 × 10^−3^) (Fig. 3B), an element that is limited in native Fogo soils (fig. S5). A PCA based on 19 elements in the leaves revealed that the loss of IRT1 in a Santo Antão background strongly contributes to the multivariate divergence of the Fogo leaf ionome profile (Fig. 3C). Together, our results suggest that loss of IRT1 was advantageous when it arose in Fogo because it increased the level of Mn in the leaves, which was severely limiting; however, *IRT1* exerts strong pleiotropic effects, and its loss of function reduced the potential for Fe transport.

### Multiple independent segmental duplications arose at *NRAMP1*, reestablishing Fe homeostasis

**Fig. 4.**
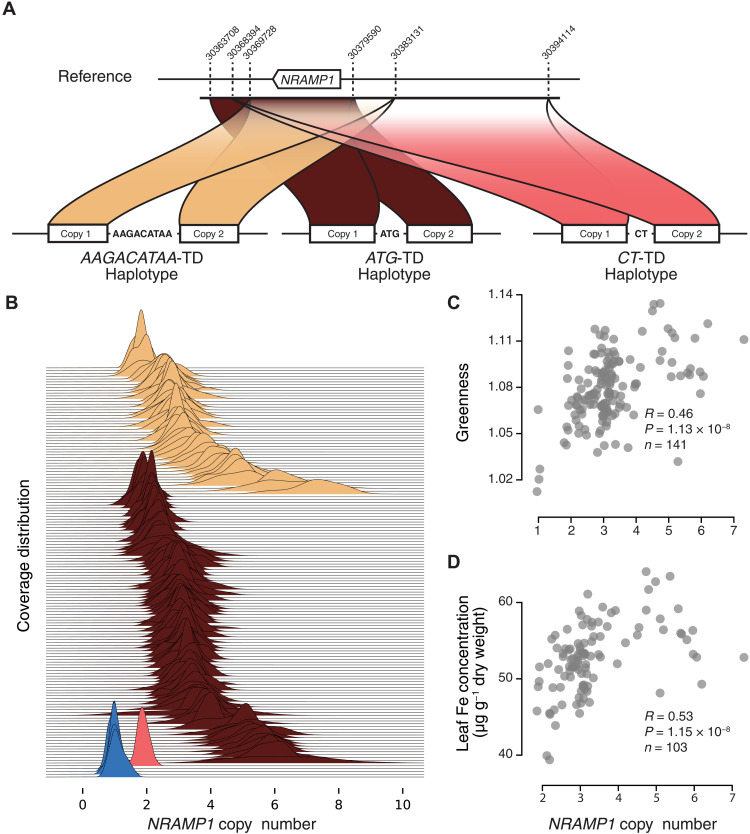
Multiple independent TDs arose at *NRAMP1*, reestablishing Fe homeostasis. (**A**) Diagram of the three independent TD events at *NRAMP1* in Fogo. Breakpoint junctions are indicated in red. Dashed lines correspond to the boundaries for each TD. The numbers above the dashed line indicate the position on chromosome 1 of the TAIR10 reference genome. (**B**) Both major *NRAMP1* haplotypes exhibit CNV across lines. Coverage distribution between the break points using 800-bp sliding windows with a step size of 400 normalized to the 3-kb surrounding regions. Haplotypes are color-coded on the basis of the schematic in (A); blue denotes plants with only a single *NRAMP1* copy. *NRAMP1* CN is positively correlated with (**C**) greenness and (**D**) leaf Fe concentration in Fogo plants. *R*, Spearman’s rho; *n*, number of measurements.

At *NRAMP1*, we found evidence for three independent tandem duplication (TD) events segregating in the Fogo population, each with distinct and nonoverlapping breakpoints in the region (Fig. 4A and S17). These TDs result from two different microhomology-mediated junctions (CT and ATG) and one 10-bp (base pair) templated junction (AAGACATAA) and are referred to below as “*CT*-TD,” “*ATG*-TD,” and “*AAGACATAA*-TD” (Fig. 4A and figs. S18 to S21). This large number of independent TD events was striking based on the short time since colonization of Fogo (~3 to 5 ka) and low SNP diversity [θ_w(Fogo)_ = 8.93 × 10^−5^]. The genomic location of *NRAMP1* may contribute to the high TD rate in the region as this gene is located in the subtelomeric region (~50 kb from the telomere), where recombination rate and thus the probability of TD events may be increased (*29*). However, we found no TDs in or around *NRAMP1* in Santo Antão accessions (*15*) and very little evidence in previously sequenced worldwide *A. thaliana* genomes (*25*–*28*). Only two accessions harbored any evidence for TDs in the region, and only one of these (in Oua-0) shows what appears to be an intact TD containing the entire *NRAMP1* coding sequence (fig. S22), indicating that if TD rates in this subtelomeric region are high in general, these are normally subject to strong purifying selection. Overall, our results indicate that the presence of three independent TD events at *NRAMP1* in Fogo is extremely unusual.

Several studies have found evidence that gene CNV plays an important role in evolution (*30*–*32*). To estimate the *NRAMP1* copy number (CN) across Fogo accessions, we first analyzed the coverage between the breakpoints compared to the surrounding region and then confirmed a subset of these using quantitative digital polymerase chain reaction (PCR) analysis (Fig. 4B, fig. S23, and table S11). We also generated whole-genome de novo assemblies for five additional Fogo genomes to further validate the breakpoint junctions and CN estimates from short-read sequencing data (fig. S24). Together, our results showed that the common *ATG*-TD and *AAGACATAA*-TD haplotypes varied in *NRAMP1* CN from two to seven, and the single *CT*-TD haplotype had two *NRAMP1* copies (fig. S23 and table S12). We also identified four Fogo accessions, all from a single stand (F10-13), with the ancestral *NRAMP1* haplotype (single *NRAMP1* copy).

We next investigated the impacts of *NRAMP1* CN on expression of the gene and physiological traits. We grew seedlings on medium and collected roots to assay *NRAMP1* expression using probe-based quantitative digital PCR (dPCR) analyses. Using multiple linear regression to account for treatment and IRT1 status, we found that *NRAMP1* CN largely explains variation in expression (ß_CNV_ = 1.06, *t* = 11.2, *P* < 1.83 × 10^−14^; model adjusted *R*^2^ = 0.819, *P* < 2.2 × 10^−16^) (fig. S25 and table S13). This experiment further showed that the loss of IRT1 causes an increase in *NRAMP1* expression in the presence of Mn (ß_IRT1_ = 0.823, *t* = 3.343, *P* < 1.7 × 10^−3^). We further assessed whether *NRAMP1* CN also affects physiological traits. On standard potting mix, *NRAMP1* CN in Fogo plants correlated strongly with greenness (Spearman’s rho = 0.46, *n* = 141, *P* = 1.13 × 10^−8^) (Fig. 4C), segmented rosette area (Spearman’s rho = 0.44, *n* = 141, *P* = 5.50 × 10^−8^), and relative chlorophyll content (Spearman’s rho = 0.78, *n* = 31, *P* = 2.96 × 10^−7^) (fig. S26). Among the 19 elements tested in the leaves, *NRAMP1* CN was most strongly correlated with Fe (Spearman’s rho = 0.53, *n* = 103, *P* = 1.15 × 10^−8^) (Fig. 4D and fig. S27). Further, using a mixed model association analysis (*33*) in the population sample, we found that *NRAMP1* CN explained 87.2% of the heritable variance in leaf Fe concentration (table S14). In contrast, *NRAMP1* CN had no effect on the heritable variance in leaf Mn concentration. In addition, F10-13-1, which carries only one *NRAMP1* copy, accumulated more Mn in the leaves compared to S1-1, further supporting the indirect causal effect of the loss of IRT1 on the Mn enrichment observed in the leaves of the Fogo plants (fig. S13). Together, these results indicate that CN expansion at *NRAMP1* partially compensates for Fe deficiency resulting from *IRT1* 130X in Fogo, consistent with the previously demonstrated role of *NRAMP1* on Fe uptake (*22*, *23*).

### The Fogo population rapidly expanded and spread across the island

**Fig. 5.**
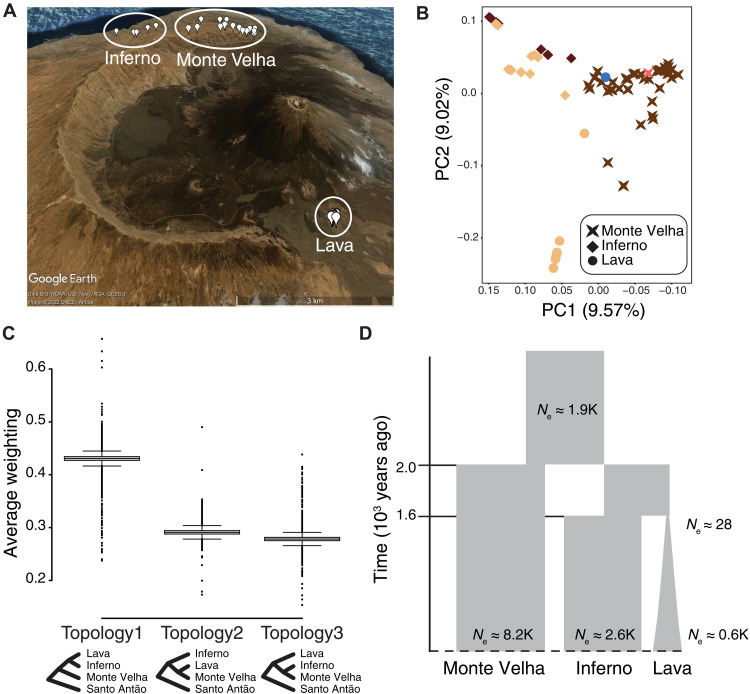
The Fogo population is strongly structured because of rapid spread across the island. (**A**) *Arabidopsis* collection locations overlaid on a satellite image of Fogo. The three major geographic regions are shown: Monte Velha, Inferno, and Lava. (**B**) Genetic structure based on the first two principal components of a linkage disequilibrium (LD)–pruned SNP matrix; color-coded with the *NRAMP1* haplotypes. (**C**) Genome-wide variation in tree topologies using Santo Antão as outgroup. Boxplots show relative weighting across the genomes. (**D**) Schematic of the inferred history of *Arabidopsis* in Fogo. *N*_e_ corresponds to effective population size.

To reconstruct the emergence and evolutionary dynamics of *IRT1* 130X and the *NRAMP1* TDs in Fogo, it is first necessary to understand the genome-wide demographic history of *Arabidopsis* in Fogo. Previously, we showed that Fogo was colonized from Santo Antão through a strong bottleneck that removed nearly all genetic variation, and that the Fogo population remained constrained in size and panmictic for ~900 years (*15*). Here, we examined relationships between subpopulations within Fogo. In PCA, we found that the genetic structure of the three major subpopulations (Monte Velha, Inferno, and Lava) closely recapitulated the geographic structure (Fig. 5, A and B, and fig. S28). To determine the order and timing of splits among the three subpopulations, we inferred their phylogenetic relationships across the genome by topology weighting (*34*). The dominant tree topology indicated that Monte Velha represents the most ancestral subpopulation, from which migrants spread into the Inferno and Lava regions (Fig. 5C and fig. S29). However, the two other possible tree topologies were nearly as common, creating a near polytomy and suggesting abrupt dispersal and divergence of the lineages. We next conducted modeling based on joint site frequency spectra (JSFS) between subpopulations, which also showed evidence for similar split times across the three subpopulation pairs (~1.6 to 2.0 ka) (Fig. 5D). However, the JSFS showed much higher shared variation between Monte Velha and Inferno compared to the geographically isolated Lava population, indicating a larger founding population size of the Inferno population or gene flow since the initial Monte Velha–Inferno split (fig. S30). Together, these results indicate that after remaining a geographically connected panmictic population for ~900 years, *Arabidopsis* spread rapidly across the island, fragmenting into distinct subpopulations by ~2 ka.

### A two-step adaptive walk rewired nutrient transport

**Fig. 6.**
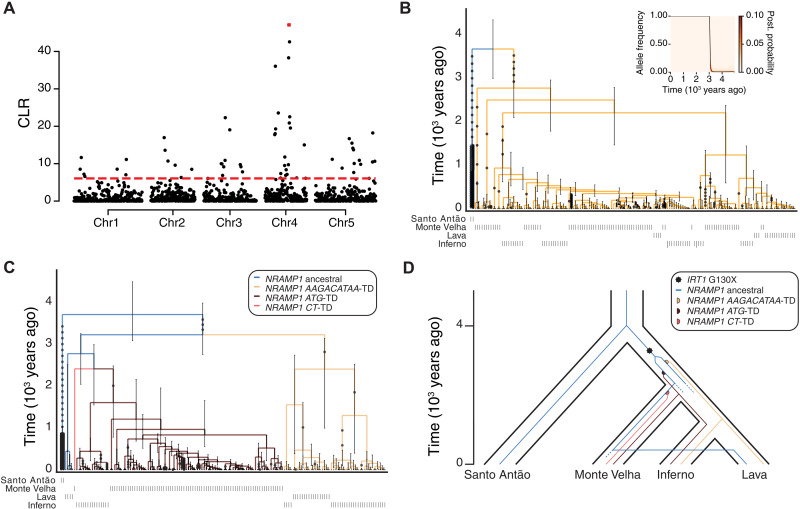
A two-step adaptive walk rewired nutrient transport. (**A**) The highest peak in a scan to identify selective sweeps in Fogo (CLR) pinpoints the *IRT1* region. CLR with nonoverlapping window of 100 kb. The red dashed line indicates the 1% tail of genome-wide CLR. The *IRT1* region is highlighted in red. (**B**) Evolutionary history of *IRT1* 130X in Fogo. In (B) to (D), the ancestral allele is colored in black. Inset: Inferred allele frequency trajectory of the derived *IRT1* 130X variant in Fogo shows evidence for rapid fixation, consistent with a hard selective sweep. The black line corresponds to the inferred allele frequency change over time and the color to the posterior probability. (**C**) Evolutionary history of *NRAMP1* TDs in Fogo. (**D**) Model of the evolutionary history of *IRT1* and *NRAMP1* in Fogo. The blue line from Monte Velha to Lava represents an apparent recent migration event.

We next examined the specific evolutionary histories of the *IRT1* and *NRAMP1* mutations. For *IRT1* 130X, fixation could have occurred by random genetic drift as a by-product of the bottleneck in Fogo. However, given the strong physiological effect on Mn levels and the very low available Mn in Fogo soils, it is also possible that the variant was fixed due to positive selection. To differentiate between selective and neutral models at *IRT1*, we applied a test for hard selective sweeps. Specifically, we used a statistic that compares the ratio of the likelihood of a sweep versus the likelihood of neutrality across the genome while accounting for local recombination rate (*35*). Overall, we found unexpectedly little evidence for hard selective sweeps across the genome (Fig. 6A), especially given the large proportion of variants inferred to be fixed by selection during the initial phase after colonization (*15*). This discrepancy may be due to the relatively short time a hard sweep signal is expected to persist in a population after fixation (*15*, *36*). The highest composite likelihood ratio (CLS) peak genome-wide localized to the *IRT1* region, where the likelihood of a sweep was ~47× greater than neutrality (Fig. 6A, fig. S31, and table S15). To infer the timing and strength of selection on *IRT1*, we conducted coalescent reconstruction of the region (*37*). We inferred that *IRT1* 130X arose ~2.76 ka [time to most recent ancestor (tMRCA); 95% confidence interval (CI): 2.09 to 3.29 ka], before the Monte Velha, Inferno, and Lava subpopulations split, and rapidly rose to fixation in Fogo (Fig. 6B and table S16). The coalescent tree for the region also provides information about the expected rate of the allele frequency change over time. From this, we estimated a selection coefficient of 13.1% for *IRT1* 130X (Fig. 6B and table S17) (*38*). Overall, the historical reconstructions at *IRT1* are consistent with a hard selective sweep acting before the Fogo population split into subpopulations.

As a group, the *NRAMP1* TD haplotypes are nearly fixed in Fogo, with a combined frequency of 98%, and they exhibit strong structure across subpopulations. Two haplotypes are common (*ATG*-TD and *AAGACATAA*-TD), while the *CT*-TD haplotype is present in a single Monte Velha individual. The single-copy *NRAMP1* haplotype is exclusive to F10-13, a population collected in the Lava region but clustering closely with Monte Velha (Fig. 5B and fig. S28). The two common haplotypes exhibit clear geographic patterns, with *ATG*-TD fixed in Monte Velha (except for the single *CT*-TD individual), *AAGACATAA*-TD fixed in Lava accessions (except the single F10-13 stand), and both haplotypes segregating at intermediate frequency in Inferno (fig. S32). Coalescent reconstruction (*37*) indicates that the two common *NRAMP1* TD haplotypes likely arose after *IRT1* 130X was fixed and after Monte Velha and Inferno subpopulations split, with estimates of ~2.38 ka (tMRCA; 95% CI: 1.07 to 3.16 ka) for *AAGACATAA*-TD and ~1.60 ka (tMRCA; 95% CI: 0.78 to 2.48 ka) for *ATG*-TD (Fig. 6C and table S16). We estimated selection coefficients of the two major *NRAMP1* haplotypes based on the trajectories of allele frequencies in the early stages (1.5 to 3.0 ka) to be 2.5 and 6.5% for the *AAGACATAA*-TD and *ATG*-TD haplotypes, respectively (table S18). Figure 6D shows a historical reconstruction of the appearance and spread of the *IRT1* and *NRAMP1* variants in the context of the population history. Multiple independent *NRAMP1* TDs arose and, as a group, reached near fixation within Fogo, likely as a result of their ability to compensate the Fe deficiency caused by the loss of IRT1. At the subpopulation scale, these appear to represent hard selective sweeps combined with some admixture, while at the island-wide scale, they represent a soft sweep, consistent with theoretical predictions (*39*, *40*).

## DISCUSSION

We showed that a two-step process rewired mineral nutrient transport in *Arabidopsis* as it colonized the volcanic island of Fogo. In the first step, an SNP that caused the truncation of the major Fe transporter, *IRT1*, exerted a strong multivariate effect on the ionome and swept to fixation in the population. In the second step, a set of independent TDs arose and reestablished Fe homeostasis by increasing CN and expression level of *NRAMP1*, which increased to near fixation by a soft selective sweep. Our results have several implications regarding evolutionary mechanisms in wild populations.

First, our results relate to the controversy over the roles of hard versus soft sweeps in adaptation (*39*–*42*). In this case, a hard selective sweep at *IRT1* appears to have occurred before the Fogo population expanded and structure developed, whereas a soft selective sweep at *NRAMP1* arose more recently, as substructure was beginning to develop on the island. This population structure would have reduced gene flow across the metapopulation and provided time for the accumulation of multiple mutations at *NRAMP1*. This is consistent with previous results showing the probability of a soft sweep from recurrent mutation centers on the chance of a second mutation arising at a locus before the initial beneficial mutation fixes in the population (*39*, *41*–*43*). Our results thus support theoretical predictions that the extent of population structure should be an important determinant of the mode of adaptation and that the definition of a hard or soft sweep may depend on the scale over which the analysis is conducted (*40*–*43*).

Second, our results provide insights regarding the architecture of adaptation. In equilibrium populations, theory predicts that most traits are complex and impacted by many small effect genetic variants (*6*, *44*). However, it has been argued that the first steps of adaptation to a novel environment should tend to have large effects (*45*). This pattern has been found in microbial populations in artificial selection experiments (*11*), but it has been less clear whether it holds in populations of multicellular organisms in the wild. This is because identifying the precise mutations that underlie adaptation and reconstructing their histories in natural populations is challenging. The pattern in Fogo is consistent with a model of rapid adaptation, where an initial large and pleiotropic evolutionary step was followed by a more moderate step, consistent with theoretical expectations of adaptation after a sudden change in the optimum (*8*).

Third, our results further support the theoretical prediction that epistasis could constrain evolution (*46*). Although IRT1 and NRAMP1 do not physically interact, they have similar functions in metal transport, and there is a delicate balance between the metal ions they transport (Fe and Mn), which are required for core photosynthetic processes (*47*, *48*). An imbalance in these metals can result in chlorosis. This could help to explain the epistatic interaction we observed with respect to the chlorotic phenotype (fig. S11). Once *IRT1* 130X was fixed in the Fogo population, the next evolutionary step was apparently severely constrained to increase CN and thus expression of *NRAMP1*.

Last, our results also have implications for sustainable agriculture. Advances in gene editing will provide new opportunities to design cost-effective specialized crops for local environments, potentially improving global food security and dietary diversity (*49*). However, to realize this potential, it is necessary to identify the genetic steps required to tailor plants to specific local conditions, including the soil environment. Here, we show that mutations in two transition metal transporters with related functions and substrates reshaped nutrient homeostasis. Overall, our results demonstrate that genetic changes at individual loci can produce substantial changes in nutrient levels in plants and thus reveal the high potential of leveraging nutrient transporters for crop improvement.

## MATERIALS AND METHODS

### Plant and sequencing materials

We phenotyped in this study accessions from Cape Verde (*15*) and Morocco (*50*) together with Cvi-0 (CS76116), Col-0 (CS76113), Col-0-*irt1* (SALK_054554C), and S1-1-*irt1.* The Col-0-*irt1* mutant was genotyped for the T-DNA insertion using the primers P1, P2, and P3 described in table S19. The S1-1-*irt1* mutant was generated in a Santo Antão background (S1-1) using CRISPR-Cas9 following the method described in (*51*). Briefly, we defined the target sites with the webtool “Knockout guide design” developed by Synthego (https://design.synthego.com/#/). Oligonucleotides containing the first 19 nucleotides of the target sites (P4, P5, P6, and P7) were used in a PCR using the pCBC-DT1T2 module vector as template. The resulting PCR product was introduced into the pHEE401E binary vector via Golden Gate cloning. Stable transgenic bacteria were selected with kanamycin (50 μg ml^−1^) and confirmed by colony PCR with the primers P8 and P9. The target sites were verified by Sanger sequencing using the primers P10 and P11. The resulting binary vectors were then transformed into *Agrobacterium tumefaciens* strain GV3101-pSOUP by electroporation. The stable transgenic bacteria were selected with rifampicin (50 μg ml^−1^), gentamicin (50 μg ml^−1^), tetracycline (5 μg ml^−1^), and kanamycin (50 μg ml^−1^). Before plant transformation, stable bacteria were grown to OD_600_ (optical density at 600 nm) of 0.7 to 1.0. The growth medium was then supplemented with 5% sucrose and Silwet L-77 (500 μl liter^−1^), and the S1-1 accession was transformed with the final binary vector following the floral dip method (*52*). We then grew the collected seeds on standard potting mix (Einheits erde Special Type Min Tray substrate) and selected a T1 chlorotic plant for further analysis. Sanger sequencing analysis at *IRT1* using the primers P10 and P11 indicated heterozygosity at the first target site (single-guide RNA1) with the absence of wild-type allele and suggested that the T1 plant was a biallelic *irt1* mutant. We then grew 60 plants of the next generation and observed chlorosis in every plant. T2 plants lacking the Cas9 transgene were identified by PCR using the primers P12 and P13 and Sanger-sequenced at *IRT1* using the primers P10 and P11. Sanger sequencing analysis indicated that two disrupting alleles of *IRT1* (*IRT1* E32fs and *IRT1* A62fs) segregated in T2 generation. In every experiment, S1-1-*irt1* corresponds to T3 seeds homozygous for the *IRT1* E32fs allele and lacking the Cas9 transgene. Every primer described here are present in table S19.

We predicted off-targets with Cas-OFFinder (*53*) using a maximum of five mismatches. We used TAIR10 as target genome and SpCas9 from *Streptococcus pyogenes* (5′-NGG-3′) as CRISPR-Cas–derived RNA-guided endonuclease. We performed whole-genome Illumina resequencing to exclude any off-targets in the two S1-1-*irt1* lines. DNA was extracted with the DNeasy Plant Mini Kit (Qiagen, catalog no. 69106). Libraries were prepared using NEBNext Ultra II FS DNA Library Prep [Illumina, New England Biolabs (NEB)]. Sequencing was performed on an Illumina HiSeq3000 platform (Illumina, NEB) with 150-bp paired-end reads to reach 50× coverage. The resulting reads were aligned to the *A. thaliana* reference genome (TAIR10) with bwa (version 0.7.15) using the mem algorithm (*54*). Duplicated reads were annotated and sorted with Picard (version 2.21.1). SNPs and indels were called with GATK HaplotypeCaller (version 4.2.3.0) using the -ERC GVCF mode (*55*), and the resulting gVCFs were merged with the parental line S1-1. We applied a filter at the position level and kept only segregating sites. Last, we examined evidence for overlap between predicted off-target sites and SNPs and indels with a custom python script (https://github.com/HancockLab/Fogo-Edaphic) and found no overlap. We used Illumina sequence data for 335 Cape Verdean accessions (*15*), 64 Moroccan accessions (*25*), 14 accessions from Madeira (*26*), 118 accessions from China (*28*), and 1135 Eurasian accessions (*27*) for multiple analyses involving population structure, selection scans, and structural variation analyses.

### Characterization of chlorosis and leaf ionome profiling on standard potting mix

To quantify the chlorosis observed in the Fogo population, we grew in four replicates 141 accessions from Fogo together with 69 accessions from Santo Antão, including Cvi-0, 62 accessions from Morocco, as well as Col-0 and S1-1-*irt1* lines. First, the seeds were stratified for 7 days at 4°C in the dark on petri dishes supplemented with 800 μl of GA4/7 (100 μM), then sown on 54-well trays (60 cm by 40 cm by 6 cm) filled with standard potting mix (Einheits erde Special Type Min Tray substrate), and fertilized with Osmocote start 11-11-7+2MgO+TE fertilizer (1 g liter^−1^). The plants were grown in controlled growth chamber conditions (8 hours of light, 21°C at day, 14°C at night). We took pictures 24 days after sowing using a Nikon D90 SLR digital camera with a Nikon DX AF-S NIKKOR 18-105 mm 1:3,5:5,6G ED lens. We next conducted image analysis using a custom Fiji (*56*) script (https://github.com/HancockLab/Fogo-Edaphic) to assess the greenness and the segmented area of each individual. Five to 6 weeks after sowing, we assessed relative chlorophyll content, quantum yield of Photosystem II, and nonphotochemical quenching using MultispeQ V2.0 following the manufacturer’s instructions.

We then harvested two to three leaves per plant for ionomic analyses. Leaf elemental content analysis was conducted using inductively coupled plasma mass spectrometry (ICP-MS) as described in (*57*). To compare the leaf ionome of the three populations, we first computed in each population a *z* score for every element extracted and removed plants with any element showing an absolute *z* score above 2. After filtering out genotypes with less than two replicates, we used the median across replicates for further ionomic analysis. Ti and Cr were used to monitor for soil contamination. As and Se were removed because the relative standard deviation (RSD) was too high. We finally combined the three populations together to conduct the PCA analysis. To add the S1-1-*irt1* on the PCA plot, we included the S1-1-*irt1* line in the Fogo population to compute the *z* score and filter out the outliers. The pipeline can be found in https://github.com/HancockLab/Fogo-Edaphic.

To characterize the effect of Fe supplementation on chlorosis in the Fogo natural accessions and the S1-1-*irt1* line, we watered the plants either with tap water only or with Fe-EDDHA (1 g liter^−1^) (Basafer Plus, Compo Expert).

### Soil characterization

We sampled soil from around *A. thaliana* plants growing on Santo Antão and Fogo islands using 20- and 50-ml Falcon tubes. Each soil sample was taken within a 20-cm radius and up to a 20-cm depth around focal plants. These samples were then subjected to ionomic analysis. We adapted a published method using water as the solvent for the elemental extraction (*58*). Soil samples were air-dried for 48 hours on weighing boats, and 5 g of each soil was weighed into 50-ml Falcon tubes. Soil samples were extracted with 25 ml of 18.2 megohm·cm of Milli-Q water (Merck Millipore) by shaking at 400 rpm in vertical position for 1 hour at room temperature. Samples were then centrifuged for 10 min at 10,000 rpm, and supernatant was collected and centrifuged again for 5 min at 14,000 rpm before sampling. The extracts were acidified with nitric acid (Primar Plus for trace metal analysis) to 2% (v/v) and analyzed with iCAP-Q ICP-MS (Thermo Fisher Scientific) equipped with collision cell technology with energy discrimination. Thirty-one elements were monitored using the following stable isotopes: ^7^Li, ^9^Be, ^11^B, ^23^Na, ^24^Mg, ^27^Al, ^31^P, ^34^S, ^39^K, ^44^Ca, ^48^Ti, ^54^V, ^52^Cr, ^55^Mn, ^56^Fe, ^59^Co, ^60^Ni, ^63^Cu, ^66^Zn, ^75^As, ^78^Se, ^85^Rb, ^88^Sr, ^95^Mo, ^107^Ag, ^111^Cd, ^133^Cs, ^137^Ba, ^205^Tl, ^208^Pb, and ^238^U. To compare the soil composition of Fogo and Santo Antão, we used the median across extractions per field site. Sample concentrations were calculated using external calibration method within the instrument software. Further data processing was performed using Microsoft Excel.

For the ionomic experiment on native soils, we used natural soils Fogo (F4—Monte Velha) and Santo Antão (S3—Espongeiro) in comparison to standard potting mix (Einheits erde Special substrate with Osmocote start 11-11-7+2MgO+TE fertilizer). For this experiment, all the pots (height = 5.5 cm, diameter = 6 cm, volume = ~100 cm^3^; Teku) were filled with 4 cm of natural soil recovered with 1 cm of the respective natural soil previously heated at 65°C in an oven for 3 days. The plants were grown in growth chamber–controlled conditions (12 hours of light, 21°C at day, 14°C at night). Five weeks after sowing, two to three young leaves were collected and washed three times with 18.2 megohm·cm of Milli-Q water (Merck Millipore) before being transferred to 1.5-ml Eppendorf tubes. The samples were then dried overnight at 80°C in an oven. To compare the leaf ionome of S1-1 and F13-8 in the different soils, we removed samples with leaf Ti concentration above 20 in μg g^−1^ of DW (ppm).

### Bulk segregant analysis

To perform BSA, we generated an F2 population by crossing the Fogo accession F13-8 to the *A. thaliana* reference accession Col-0. The F2 seeds and their corresponding parents were first stratified for 7 days at 4°C in the dark on petri dishes supplemented with 800 μl of GA4/7 (100 μM), then sown on squared pots (7 cm by 7 cm, height = 8 cm; Göttinger) with standard potting mix, and transferred to controlled greenhouse conditions (16 hours of light, 8 hours of dark, 21°C). To examine the chlorosis segregation, we took pictures of each F2 individual’s rosette 4 weeks after sowing using a Nikon D90 SLR digital camera with a Tamron SP AF 90 mm F/2.8 macro lens and ColorChecker (X-Rite, catalog MSCCC) for color control. We counted 24 highly chlorotic plants out of 454 in F2 individuals in total. DNA was extracted from two young rosette leaves of each F2 individual with the BioSprint 96 DNA Plant Kit (Qiagen, catalog no. 941557) and quantified using a Qubit fluorometer (Thermo Fisher Scientific, catalog no. Q33238). The DNA concentration was then adjusted to 1 ng μl^−1^ for each extraction, and the DNA integrity was checked on a 1% agarose gel. We next conducted image analysis using a custom Fiji (*56*) script (https://github.com/HancockLab/Fogo-Edaphic) to assess the greenness of each F2 individual. After examining the greenness distribution, we could recover nine highly chlorotic individuals for bulk sequencing and pooled 20 ng of DNA from each selected individual (Fig. 3B). Library preparation and Illumina HiSeq3000 sequencing were performed by the Max Planck Genome Center in Cologne (Germany). Briefly, the libraries were prepared using NEBNext Ultra II FS DNA Library Prep (Illumina, NEB) and sequenced with 150-bp paired-end reads to 36× coverage using the Illumina HiSeq3000 platform (Illumina, NEB).

The reads were aligned to the *A. thaliana* reference genome (TAIR10) with bwa (version 0.7.15) using the mem algorithm (*54*). Duplicated reads were annotated and sorted with picard (version 2.21.1). SNPs and short indels were called with GATK HaplotypeCaller (version 4.1.3.0) (*55*) using the reference confidence model (BP_resolution mode). We filtered the variants with a custom Python (version 2.7.13) script to keep only the positions with a genotype quality GQ > 25, a total depth DP > 3, and no missing allele in the corresponding parents. In addition, we filtered out variants with minor allele frequencies below 5% in the Fogo population. To identify candidate genes after BSA, we annotated with SnpEff (version 4.3r) (*59*) the variants present in windows where the Col-0 allele frequency was above 0.99 for chromosome 1 and below 0.01 for chromosome 4. We then extracted the predicted high-impact variants present in F13-8. For chromosome 1, since we did not identify strong candidates based on SNP and short indel variation, we examined the presence of structural variation in F13-8 using Delly (version 0.8.3) (*60*) and annotated them using SnpEff (version 4.3r) (*59*). The pipeline can be found in https://github.com/HancockLab/Fogo-Edaphic. Multiple alignments were produced with BoxShade (https://sourceforge.net/projects/boxshade/) based on the genomic variant calls at *IRT1* and *NRAMP1* (*15*).

### F2 association

To follow up the results from BSA, we further examined segregation of specific candidate variants in the F13-8 × Col-0 F2 population. We genotyped these by derived cleaved amplified polymorphic sequences (dCAPS) on each F2 individual and five replicates per parental line as controls. We genotyped SNPs lying within the coding region of *IRT1* (Chr4: 10 707 874) and *NRAMP1* (Chr1: 30 374 389) by using the restriction enzymes Dde I (NEB, catalog no. R0175S) and Sac I–HF (NEB, catalog no. R3156L), respectively. We used dCAPS finder 2.0 (http://helix.wustl.edu/dcaps/dcaps.html) to generate the PCR primers (P14 and P15 for *IRT1*, P16 and P17 for *NRAMP1*). The polymorphic regions were PCR-amplified in a 25-μl reaction containing 15 μl of dH_2_0, 0.5 μl of dNTPs (10 mM), 1 μl of forward and reverse primer (10 μM each), 2.5 μl of MgCl_2_ (25 mM) (Bio-Budget, catalog no. 80-60010100), 2.5 μl of 10× BD buffer (Bio-Budget, catalog no. 80-60010100), 0.5 μl of Taq polymerase (Bio-Budget, catalog no. 80-60010100), and 2 μl of genomic DNA. Ten microliters of the corresponding PCR products was digested for 3 hours at 37°C with 2 μl of 10× CutSmart buffer, 0.5 μl of appropriate restriction enzyme, and 7.5 μl of H_2_0. The resulting digested products were visualized on a 3.5× agarose gel. Primers described here are detailed in table S19.

### Characterizing variation in the *NRAMP1* region from short-read data

We used Delly (version 0.8.3) (*60*) to genotype the region surrounding *NRAMP1* for the presence or absence of a TD in short-read-sequenced genomes from Morocco (*25*, *50*), Eurasia (*27*), and Cape Verde (*15*). We confirmed every TD manually in the Integrative Genomics Viewer (IGV) (*61*). The pipeline can be found in https://github.com/HancockLab/Fogo-Edaphic.

### Whole-genome assembly

The seeds intended for Nanopore sequencing were stratified for 7 days at 4°C in the dark on petri dishes before sowing on pots (7 cm by 7 cm, height = 8 cm; Göttinger) containing standard potting mix. Five seedlings per pot were left and grown under greenhouse conditions (16 hours of light, 8 hours of dark, 21°C) for 3 weeks. Library preparation and Nanopore sequencing were performed by the Max Planck Genome Center in Cologne (Germany). Briefly, the genomic DNA was extracted from pooled leaf tissue from the same genotype. DNA fragments above 30 kb were selected with BluePippin (Sage Science). The libraries were prepared using Ligation Sequencing Kit 1D (Oxford Nanopore Technologies, catalog no. SQK-LSK109) and sequenced using the GridION X5 platform (Oxford Nanopore Technologies).

De novo assemblies were generated with miniasm-0.3 (*62*) and minimap2-2.17 (*63*). The resulting draft assemblies were first polished twice with racon (version 1.4.10) (*64*) using the raw Nanopore reads and then 10 more times with pilon (version 1.23) (*65*) using the corresponding Illumina short reads. We oriented and scaffolded the contigs using the reference genome assembly (TAIR10). To prevent mapping of Illumina short reads to anchoring points of the contigs, we inserted 1000 N’s between the contigs. The pipeline can be found in https://github.com/HancockLab/Fogo-Edaphic.

### *NRAMP1* CN estimation

To estimate *NRAMP1* CN based on short-read sequenced genomes, we first extracted the breakpoint positions using Delly (version 0.8.3) (*60*). We then obtained the coverage at each base pair between the positions 303,5000 and 304,0000 on chromosome 1 using the “--depth” command in VCFtools (version 0.1.16) (*66*). We next used a Python (version 3.8.3) custom script to calculate the coverage in 800-bp sliding windows every 400 bp and normalized it to the 3-kb regions upstream and downstream of the breakpoints. For the single *NRAMP1* copy, since no breakpoints were found, we use the most extreme breakpoint positions found at *NRAMP1* in Fogo (start: 30363708; end: 30394114). We then used the median of each ratio to test the association between *NRAMP1* CN and the different phenotypes. The pipeline can be found in https://github.com/HancockLab/Fogo-Edaphic.

To assess *NRAMP1* CN by dPCR, we first extracted genomic DNA from leaf tissue with the DNeasy Plant Mini Kit (Qiagen, catalog no. 69106) and quantified it using the Qubit fluorometer (Thermo Fisher Scientific, catalog no. Q33238). The dPCRs were performed with the QIAcuity 5-plex device (Qiagen, catalog no. 911021) in 26K (24-well) Nanoplates (Qiagen, catalog no. 250001). We chose Eco RI–HF for DNA fragmentation after confirming the absence of Eco RI–HF site at the DNA targets. The dPCR mixtures were set up with the QIAcuity EG PCR Kit (Qiagen, catalog No. 250111) following the manufacturer’s instructions with 5 ng of genomic DNA together with 0.2 U of Eco RI–HF (NEB) per reaction and left for 15 min at room temperature for DNA fragmentation. The thermal cycling conditions were as follows: initial heat activation, 2 min at 95°C; three-step cycling conditions (40 cycles), 15 s at 95°C, 15 s at 55°C, and 15 s at 72°C; cooling down, 5 min at 40°C. Absolute quantification and CNV were estimated with QIAcuity Software Suite 1.2.18 (Qiagen). EvaGreen detection was done using the green channel with 100-ms exposure and gain 6. We chose *PHOSPHATASE 2A (PP2A)* (AT1G13320) as a reference gene after excluding the presence of extra copies with IGV (*61*). We used the primers P18 and P19 for *NRAMP1* and P20 and P21 for *PP2A* for the dPCRs. Every primer described here is detailed in table S19.

To estimate the number of *NRAMP1* copies based on long-read sequenced genomes, we aligned all assembled genomes individually to the reference genome assembly (TAIR10) with the nucmer package from MUMmer (version 3.1) (*67*) with the following parameters: --maxmatch, -l 100, -c 500. We then identified structural variants using Assemblytics (version 1.2) (*68*) with the following parameters: unique sequence length required, 1000 bp; maximum variant size, 100,000 bp; and minimum variant size, 50 bp. The pipeline can be found in https://github.com/HancockLab/Fogo-Edaphic.

### *NRAMP1* expression analysis

To quantify mRNA levels in root tissues, seedlings were grown under sterile conditions on vertically oriented square 120-mm polystyrene petri dishes (Greiner). After surface sterilization using sodium hypochlorite solution (40%), the seeds were stratified for 7 days at 4°C in the dark and transferred to a growth chamber (Percival Scientific) in long-day condition (16 hours of light, 8 hours of dark, 21°C). Total RNA extraction was performed using TRIzol (Invitrogen, catalog no. 15596026). One microgram of total RNA was treated with the DNA-free DNA Removal Kit (Invitrogen, catalog no. AM1906) for 1 hour at 37°C following the manufacturer’s instructions. Complementary DNA was generated using the SuperScript II reverse transcriptase (Invitrogen, catalog no. 18064022) together with oligo(dT)18 primer for 2 hours at 42°C.

To compare the expression of *NRAMP1* between F13-8 and Col-0, we used a (1×) Murashige and Skoog (MS) medium and harvested root tissue from 15 plants 12 days after the shift to long-day conditions. mRNA levels were assessed by quantitative PCR on a LightCycler 480 instrument (Roche) using the EvaGreen dye (Biotium, catalog no. 31000) and the my-Budget Taq DNA polymerase (Bio-Budget, catalog no. 80-60010100). We used the primers P22 and P23 to detect *NRAMP1* mRNA and the primers P20 and P21 to detect *PP2A* mRNA. We applied the −∆∆*C*_t_ method with Pfaffl correction using *PP2A* (AT1G13320) as reference gene and S1-1 as control genotype.

To assess the expression of *NRAMP1* in response to different Mn concentrations, we cultivated seedlings for 7 days in a Hoagland medium solidified with 0.8% (w/v) agar type M (Sigma-Aldrich) and containing 1% (w/v) sucrose and 10 μM FeHBED (*69*). We then transferred the seedlings onto a fresh modified Hoagland medium, which was solidified with EDTA-washed 0.8% (w/v) agar type M to remove any contaminant metals, and which contained 10 μM FeHBED and either 10 μM Mn (+Mn) or no Mn (−Mn). We harvested root tissue from 15 14-day-old plants. mRNA levels were quantified by dPCR using the QIAcuity five-plex device (Qiagen, catalog no. 911001) in QIAcuity Nanoplates 26K 24-well plates (Qiagen, catalog no. 250001). The dPCR mixtures were set up in duplex with the QIAcuity Probe PCR Kit (Qiagen, catalog no. 250101) following the manufacturer’s instructions. We used the primers P25 and P26 with the probe P26 (5′ FAM, BHQ1 3′) for *NRAMP1* and the primers P20 and P21 with the probe P27 (5′ ROX, BHQ2 3′) for *PP2A*. For normalization, we used *PP2A* as reference gene and Col-0 in +Mn as control genotype. Primers and probes used in this study are detailed in table S19.

### Heritability

We estimated the heritable variance in Fe and Mn concentrations with GEMMA (version 0.94) (*33*). We applied a linear mixed model that accounts for population structure by including a kinship matrix in the model. As previously described (*15*), we used a VCF file generated with GATK. This VCF file was filtered with VCFtools (version 0.1.16) (*66*) to consider only biallelic positions and individuals having a genotype quality above 25 (GQ > 25) and a total depth above 3 (DP > 3). We then used *NRAMP1* CN estimates as a covariate.

### Ionomics on plates

To quantify metal concentrations in leaf and root tissues, seedlings were cultivated under sterile conditions on vertically oriented square 120-mm polystyrene petri dishes (Greiner Bio-One) in 16-hour light (22°C, 120 μmol m^2^ s^−1^, white light)/8-hour dark (18°C) cycles in a growth chamber (Percival CU-41L4; CLF Climatics). After surface sterilization for 1 min in 70% (v/v) EtOH, followed by 10 min in 1.4% (v/v) NaOCl and 0.02% (v/v) Triton X-100, and four final washing steps in sterile water, 40 seeds per plate were transferred onto modified Hoagland medium containing 1% (w/v) sucrose and solidified with 0.8% (w/v) agar type M (Sigma-Aldrich) (*69*). Col-0 and Col-0-*irt1* were stratified at 4°C in the dark for 2 days, whereas media for all other genotypes were additionally supplemented with 100 μM GA4+7 (Duchefa), followed by stratification for 7 days. After precultivation for 10 days, seedlings were transferred onto fresh modified Hoagland medium containing 10 μM FeHBED and solidified with EDTA-washed 0.8% (w/v) agar type M, from which contaminant metals had been removed by washing in EDTA solutions (*69*). Pools of leaf and root tissues of 15 to 30 17-day-old seedlings were harvested from one or two plates by cutting below the hypocotyl using a scalpel. Desorption to remove extracellularly bound metal cations was conducted in 50 ml of 5 mM CaSO_4_, 1 mM MES-KOH (pH 5.7) for 10 min, 50 ml of 5 mM CaSO_4_, 10 mM Na_2_EDTA, and 1 mM MES-KOH (pH 5.7) for 5 min, followed by two washes in 50 ml of ultrapure water for 1 min, all conducted on ice with occasional stirring. Desorbed tissues were blotted dry using paper towels (Blauer Engel) and dried in paper bags at 60°C for 3 days. After 3 days of equilibration to room temperature, plant material was weighed and acid-digested as in (*70*). Multi-element analysis was conducted using inductively coupled plasma optical emission spectrometry (ICP-OES) in an iCAPDuo 6500 instrument (Thermo Fisher Scientific). Calibration was done with a blank and a series of five multielement standards from single-element standard solutions (AAS Standards, Bernd Kraft), with QC (quality control) using a sample blank and an intermediate calibration standard solution, as well as digests of certified reference material (Virginia tobacco leaves, INCT-PVTL 6; Institute of Nuclear Chemistry and Technology, PL).

### Population structure and migration history of the Fogo populations

To examine population structure, the original VCF (*15*) was first filtered using bcftools (version 1.9) (*71*) with the command “bcftools view -m2 -M2 -v snps -i ‘MIN(FMT/DP)>3 & MIN(FMT/GQ)>25 & F_MISSING = 0.” The resulting biallelic SNPs were then pruned for short-range linkage disequilibrium (LD) by removing any SNP that showed correlation coefficients (*r*^2^) greater than 0.1 in a window size of 50 kb and step size of 10 bp. We conducted PCA using the --pca option in PLINK (version 1.9) (*72*).

We quantified the evolutionary relationships among subtrees of the Fogo populations using Twisst (*34*) with the Santo Antão population as outgroup. Biallelic SNPs were first converted to “.geno” format using the script “parseVCF.py” (https://github.com/simonhmartin/genomics_general/tree/master/VCF_processing). Subsequently, we generated maximum-likelihood trees in sliding windows of 50 SNPs with the BIONJ (*73*) algorithm in PhyML (version 3.9_360-500M) (*74*) using the script “phyml_sliding_windows.py” (https://github.com/simonhmartin/genomics_general/tree/master/phylo). We ran Twisst on the inferred trees using the “--complete method” option. The pipeline can be found in https://github.com/HancockLab/Fogo-Edaphic.

We used custom scripts to compute folded JSFS (https://github.com/HancockLab/Fogo-Edaphic) across each pair of genetic groups in Fogo, namely, Monte Velha–Inferno, Monte Velha–Lava, and Inferno-Lava. The JSFS were computed on the entire genome after excluding sites with more than 5% missing data, CpG sites, due to their hypermutable nature, pericentromeric regions, which are rich in satellite repeats, and other repeat regions identified with Heng Li’s SNPable approach (http://bit.ly/snpable). For each SNP that passed quality filtering, and each genetic cluster, we took a random set of 90% of the accessions with no missing data at the focus SNP to generate the JSFS.

We inferred demographic histories of the three Fogo subpopulations (Monte Velha, Inferno, and Lava) by fitting models to the JSFS using ∂a∂i (version 2.1.0) (*75*). We used two sets of models: First, we optimized parameters for four two-population models applied to all pairwise combinations of the three populations. The models are as follows: (i) a population split with no migration and constant population size (Ne); (ii) a population split with a single change in Ne, which can converge toward a bottleneck; (iii) a split followed by exponential changes in Ne and no migration; and (iv) an isolation with migration model (IM): a split followed by exponential changes in Ne and asymmetric migration.

We ran ∂a∂i in 200 independent replicates with a maximum of 50 iterations, for each model and each combination of populations. In each run, we randomly drew starting values for parameters within standard parameter boundaries: (10^−3^ × *N*_ref_; 20 × *N*_ref_) for effective population sizes (Ne), (0; 20/*N*_ref_) for migration rate, and (0; 10 × *N*_ref_) for split times, where *N*_ref_ is the size of the ancestral population. Within each model, among the 200 independent runs, we selected the parameter set that resulted in the highest likelihood. Then, we compared support across models with the Akaike information criterion (AIC). We assumed a generation time of 1 year and a mutation rate of 7.1 × 10^−9^ based on estimates from mutation accumulation lines (*76*).

### Geographical maps

For the geographical distribution of the natural populations in Fogo, we used Google Earth: Image 2021 CNES/Airbus, Data SIO, NOAA, U.S. Navy, NGA, GEBCO. We used R (version 3.6.2) for the geographical distribution of the *NRAMP1* haplotypes in Fogo. The pipeline can be found in https://github.com/HancockLab/Fogo-Edaphic.

### Inferences of selection

For the inference of genomic signatures of selective sweeps, we used SweepFinder2 (*35*) with a grid size of 100 kb. We used a recombination map inferred from crosses (*77*), and we corrected it for the rate of outcrossing inferred from natural populations (*78*). Because the Fogo population evolved in isolation from the continents after a colonization bottleneck, we polarized the site frequency spectrum to the reference genome, TAIR10. We considered genomic regions with the highest percentile of the CLR distribution as having the strongest signatures of selection. To define the borders of the selective sweep region overlapping with *IRT1*, we examined the successive 100-kb windows above the 1% percentile of the genome-wide CLR (18.28) and obtained a 502,269-bp region (Chr4: 10548683 and Chr4: 11050953). We then annotated variants present within this region and private to the Fogo population using SnpEff (version 4.3r) (*59*).

### Reconstruction of evolutionary histories of *IRT1* and *NRAMP1* loci in Fogo

We inferred the genealogical trees for the derived alleles of *IRT1* and *NRAMP1* with Relate (version 1.1.4) (*37*). Then, we inferred historical frequency trajectories and selection coefficients for *IRT1* 130X (Chr4: 10707974) and the *NRAMP1* TDs (Chr1:30363708 and Chr1:30369728) using importance sampling over local genealogies in CLUES (*38*). To produce genealogies, we used as the outgroup S1-1 and S8-505, two accessions that originated from the Santo Antão subpopulation that is genetically closest to the Fogo population based on clustering in an NJ tree (*15*). We used bcftools (version 1.9) (*71*) to extract from the VCF file biallelic SNPs with the command “bcftools view -U -c 1 -m2 -M2 -v snps” and to remove any position where at least one individual did not pass the filter quality with the command “bcftools view DP > 3 MIN(FMT/DP)>3 &MIN(FMT/GQ)>25 & F_MISSING=0.” For *NRAMP1*, we introduced variants into the “hap” file corresponding to the break point junctions as shown in (Fig. 5A). We ran Relate (*37*) under a haploid model using a mutation rate parameter based on the spontaneous mutation rate previously estimated in *A. thaliana* (*76*), corrected for missing data for each region with the formula $7\times 10^{-9}\times (1-\frac{\text{missing variants}}{\text{total variants}})$. We obtained 1.55189 × 10^−9^ for *IRT1* G130X (Chr4: 10707974) and 1.42794 × 10^−9^ for *NRAMP1* TDs (Chr1: 30363708 and Chr1: 30369728). For the “--map” parameter, we used a previously described recombination map (*77*) and divided the genetic distances by 20 to correct for the outcrossing rate of 5% estimated in natural populations (*78*). We set the generation time to 1 year “—year_per_gen 1,” sampled 200 branch lengths with the “SampleBranchLength.sh” script, and plotted the trees of interest using the “TreeViewSample.sh” script.

Selection coefficients were inferred by comparing the local genealogies against the genome-wide coalescence rates (--coal) inferred previously (*15*), which provides conservative estimates for the local trees against background population growth. We obtained estimates of the posterior distributions of allele frequencies over time using a recessive model for *IRT1* 130X (-- dom 0): <inference.py --popFreq 0.99 --tCutoff 5000 --coal relate.popsize.coal --sMax 1 –df 100 --dom 0> and an additive model for the *NRAMP1* TDs (-- dom 0.5): <inference.py --popFreq 0.99 --tCutoff 5000 --coal relate.popsize.coal --sMax 1 –df 100 --dom 0.5>. We inferred selection coefficients jointly across time bins of 1.5 ka between the present day and the time in the past when the variants arose. We used three-time bins (epochs) (0 to 1.5, 1.5 to 3, and 3 to 4.5 ka) for *IRT1* 130X (table S17) and two-time bins (0 to 1.5 and 1.5 to 3 ka) for *NRAMP1* TDs (table S18). The pipeline can be found in https://github.com/HancockLab/Fogo-Edaphic.
