# Supplementary material for: A two-step adaptive walk rewires nutrient transport in a challenging edaphic environment
Source: Sci Adv. 2022 May 18;8(20):eabm9385. doi: 10.1126/sciadv.abm9385 (PMC9116884; doi:10.1126/sciadv.abm9385)
Supplement: Supplementary file 1 — Figs. S1 to S32 [file sciadv.abm9385_sm.pdf]

Science Advances
AAAS

Supplementary Materials for

A two-step adaptive walk rewires nutrient transport in a challenging edaphic environment

Emmanuel Tergemina*, Ahmed F. Elfarargi, Paulina Flis, Andrea Fulgione, Mehmet Göktay, Célia Neto, Marleen Scholle, Pádraic J. Flood, Sophie-Asako Xerri, Johan Zicola, Nina Döring, Herculano Dinis, Ute Krämer, David E. Salt, Angela M. Hancock*

*Corresponding author. Email: hancock@mpipz.mpg.de (A.M.H.); tergemina@mpipz.mpg.de (E.T.)

Published 18 May 2022, *Sci. Adv.* **8**, eabm9385 (2022)
DOI: 10.1126/sciadv.abm9385

The PDF file includes:

Figs. S1 to S32
Legends for tables S1 to S19

Other Supplementary Materials for this manuscript includes the following:

Tables S1 to S19

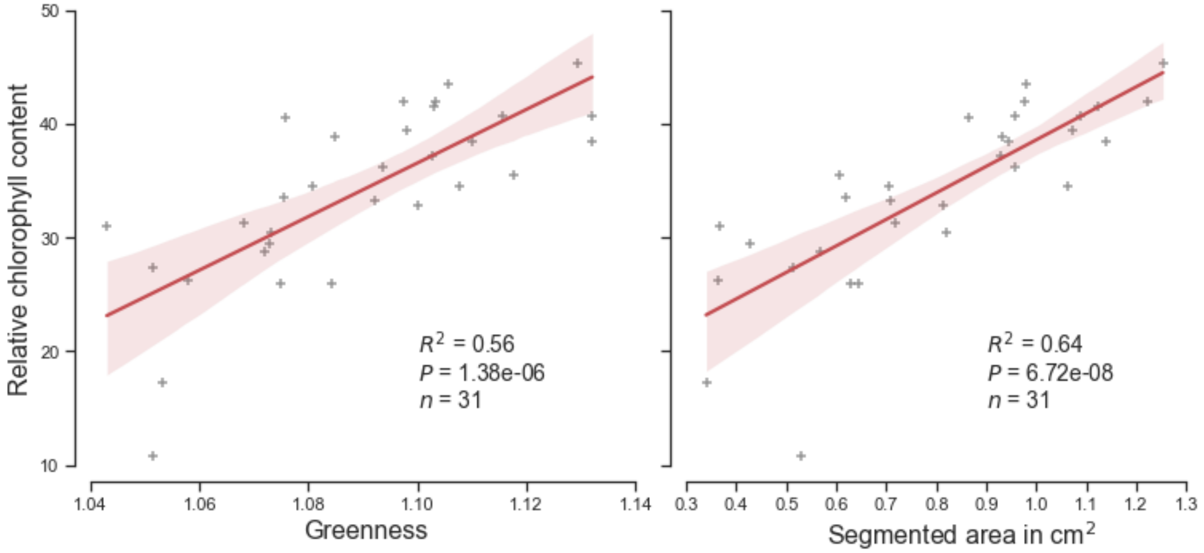

Fig. S1. Relative chlorophyll content correlates with chlorosis observed by image analysis in a representative subset of Fogo population.

Linear correlation of the relative chlorophyll content and the greenness and the segmented area in cm^2 in a representative set of 31 accessions from Fogo. R^2 = Pearson's R^2, P = p-value and n = number of genotypes. Pictures were taken 24 days after sowing. Relative chlorophyll content was evaluated with the multispeQ tool 5 to 6 weeks after sowing. Each dot represents the median across 3 replicates per accession.

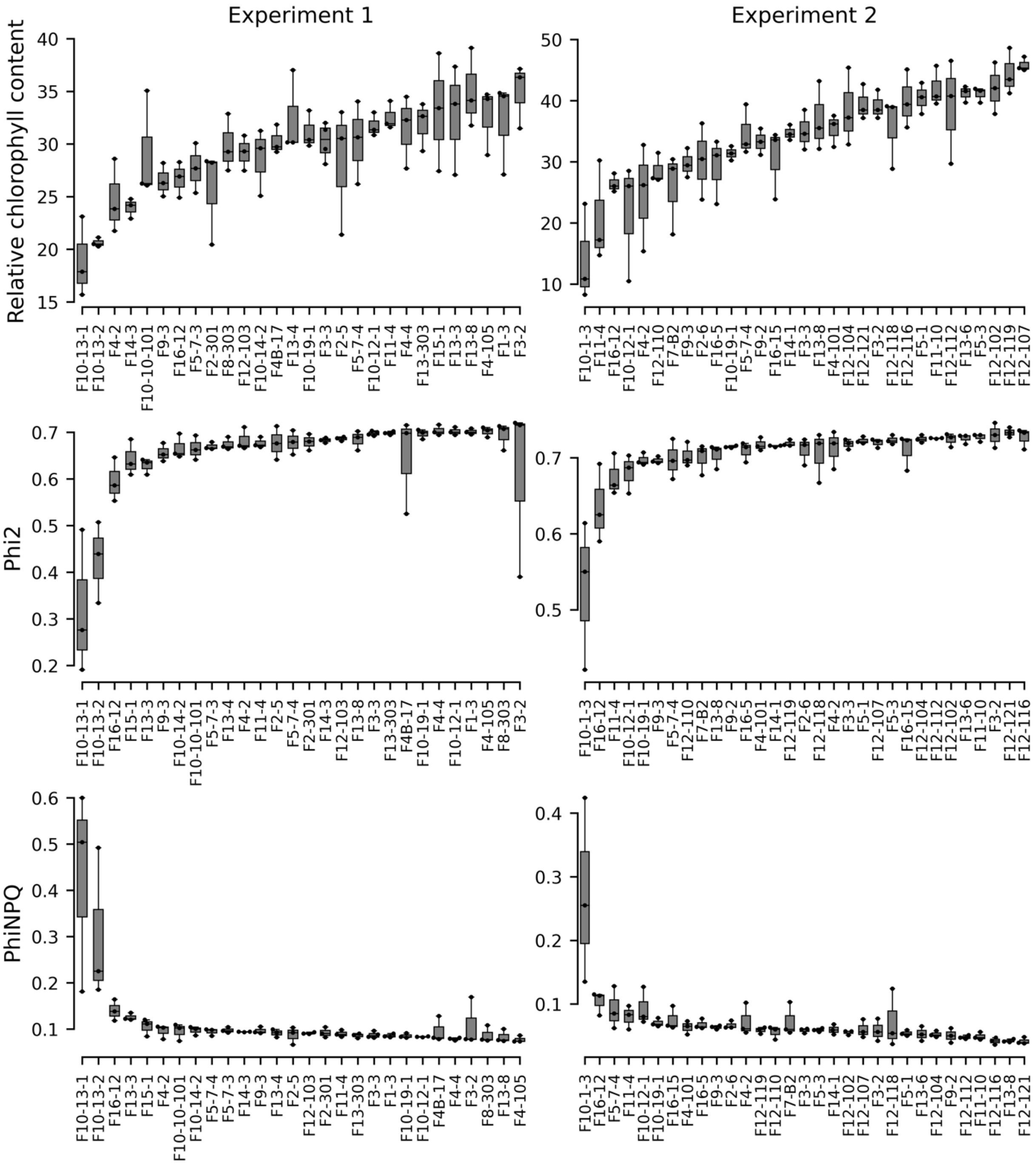

Fig. S2. Variation in photosynthetic activity in the Fogo population.

Variation in relative chlorophyll content, quantum yield in Photosystem II (Phi2) and non-photochemical quenching (PhiNPQ) in two different experiments. The plants were grown in standard potting mix and greenhouse conditions (12 hours of light, 21 ºC at day, 14 ºC at night). The measurements were taken at bolting. Each dot represents one replicate per genotype.

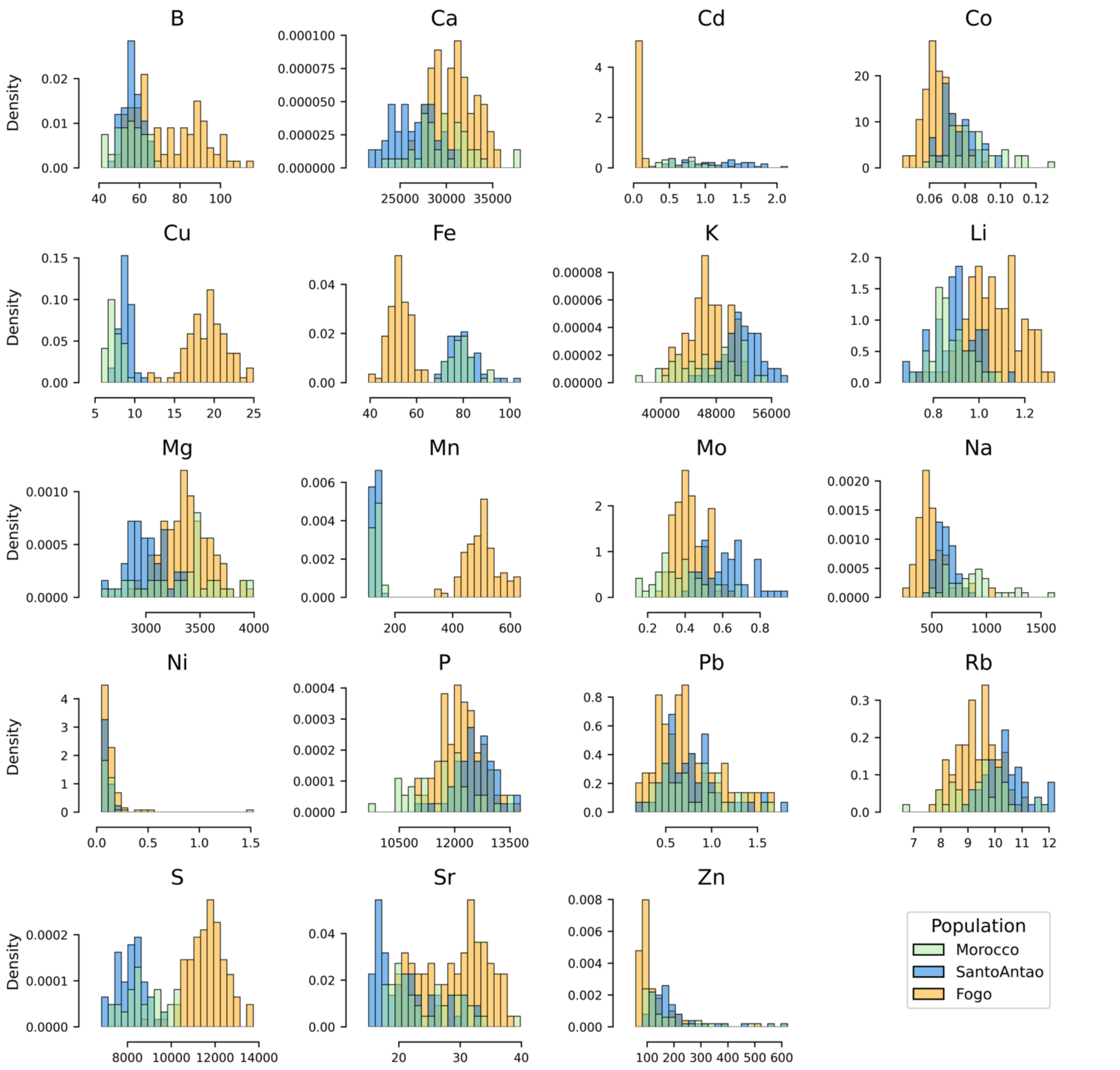

Fig. S3. Variation in leaf elements accumulation in the Fogo population relative to Santo Antão and Morocco.

Distribution of 19 elements in the leaves of Fogo (orange), Santo Antão (blue) and Morocco (green) plants. The plants were grown on standard potting mix in controlled growth chamber conditions (12 hours of light, 21 ºC at day, 14 ºC at night, 70% humidity). The values on the x axis are indicated in $\mu g \bullet g^{-1}$ of dry weight (ppm) and correspond to the median across replicates. The tissue was harvested five to six weeks after sowing.

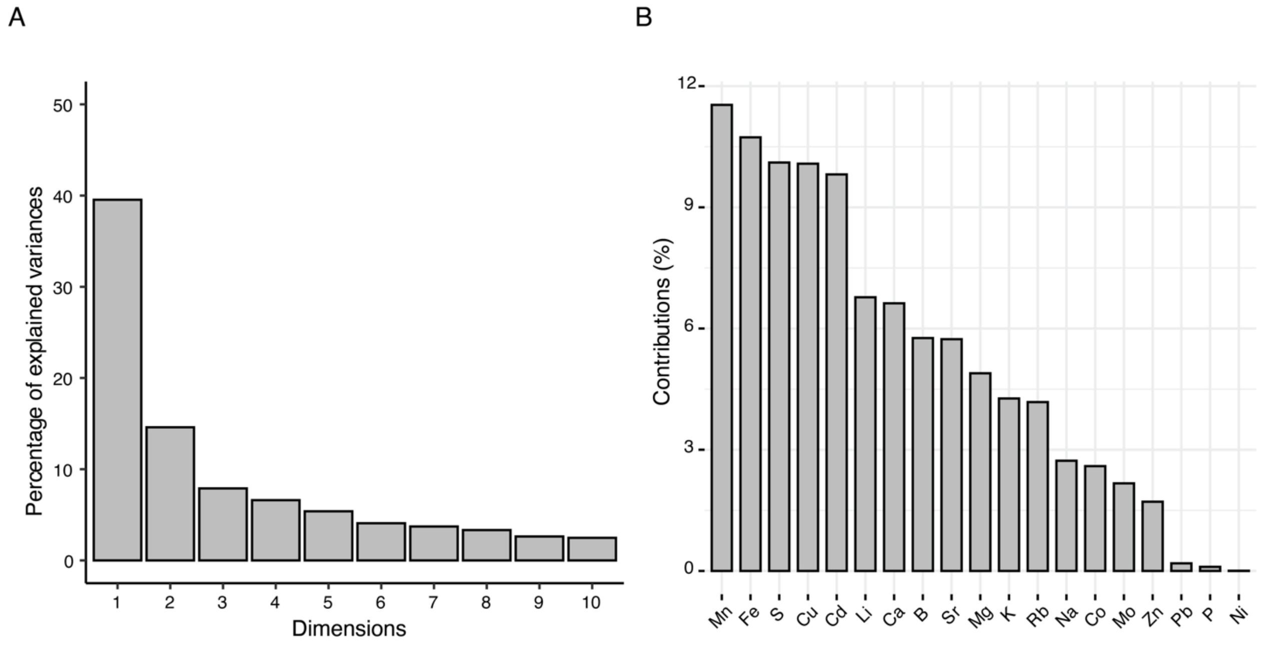

Fig. S4. Scree plot of 10 PCA eigenvalues and the contribution of leaf concentration of 19 elements to PC1.

(**A**) Contributions of the 10 first principal components on the leaf ionome of Fogo, Santo Antão and Morocco plants based on 19 elements. (**B**) Contributions of the 19 elements on the first principal component.

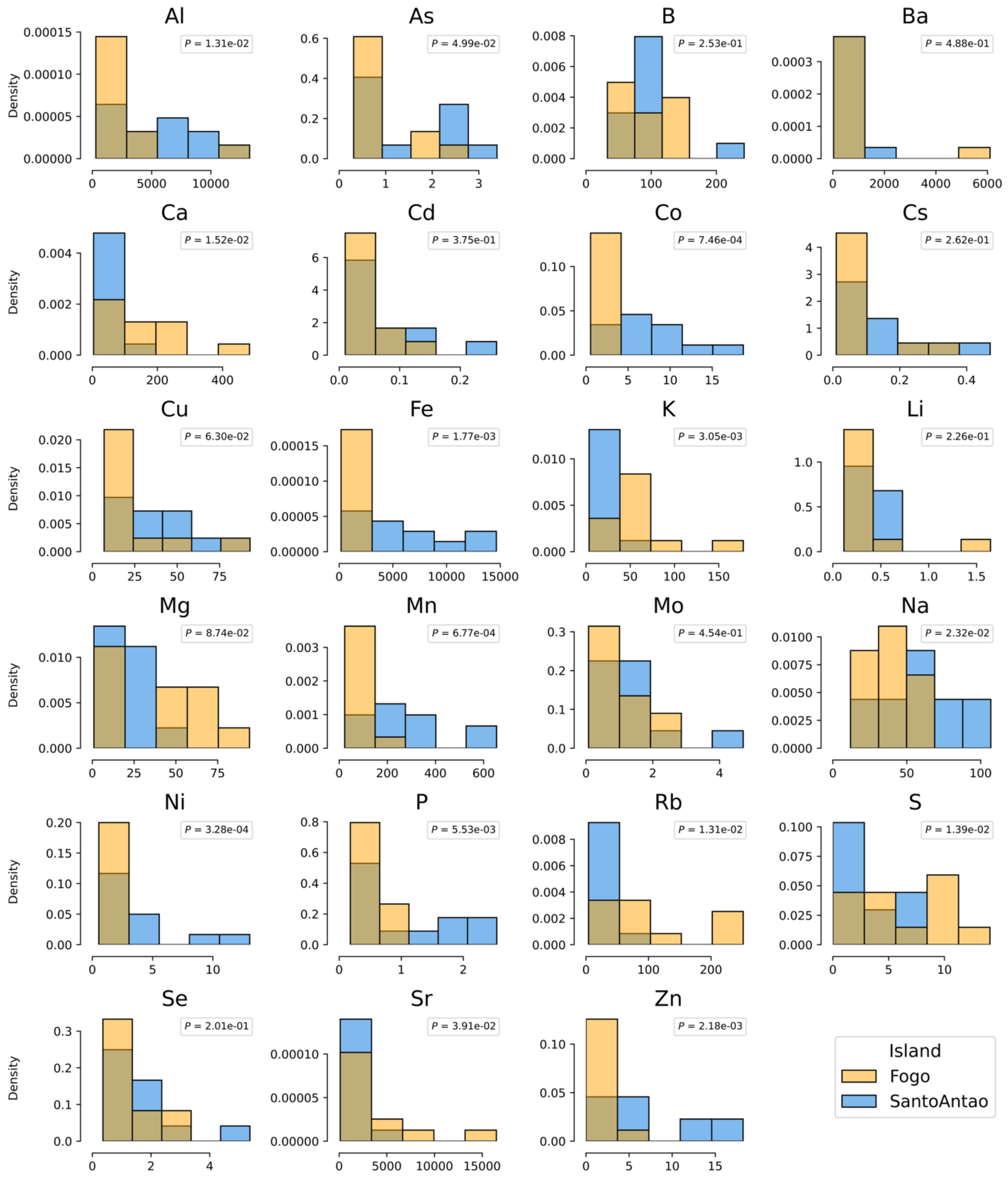

Fig. S5. Soil characteristics of Fogo and Santo Antão.

Distribution of 23 elements in topsoils from Fogo in orange and Santo Antão in blue. Values on the x axis are shown in ng•g^{-1} of dry weight (ppb) except for Na, Mg, Ca, S, K and P indicated in µg•g^{-1} of dry weight (ppm); and correspond to the median across extractions per field site. The extractions were done with water. P = p-value for Mann-Whitney-Wilcoxon test.

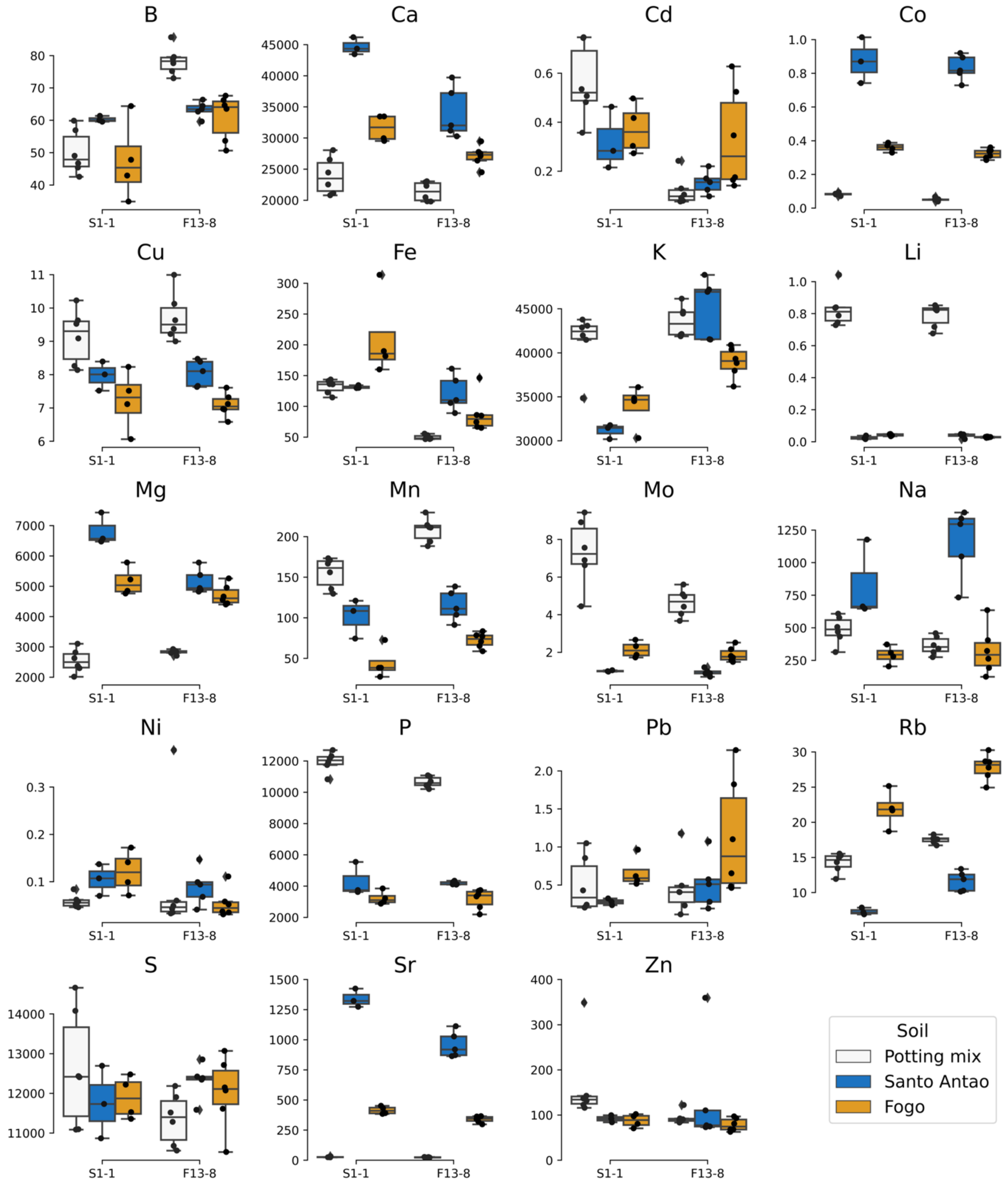

Fig. S6. Leaf element accumulation in S1-1 and F13-8 grown on native soils and standard potting mix.

Values are shown in $\mu g \bullet g^{-1}$ of dry weight (ppm). Each dot represents one replicate per genotype.

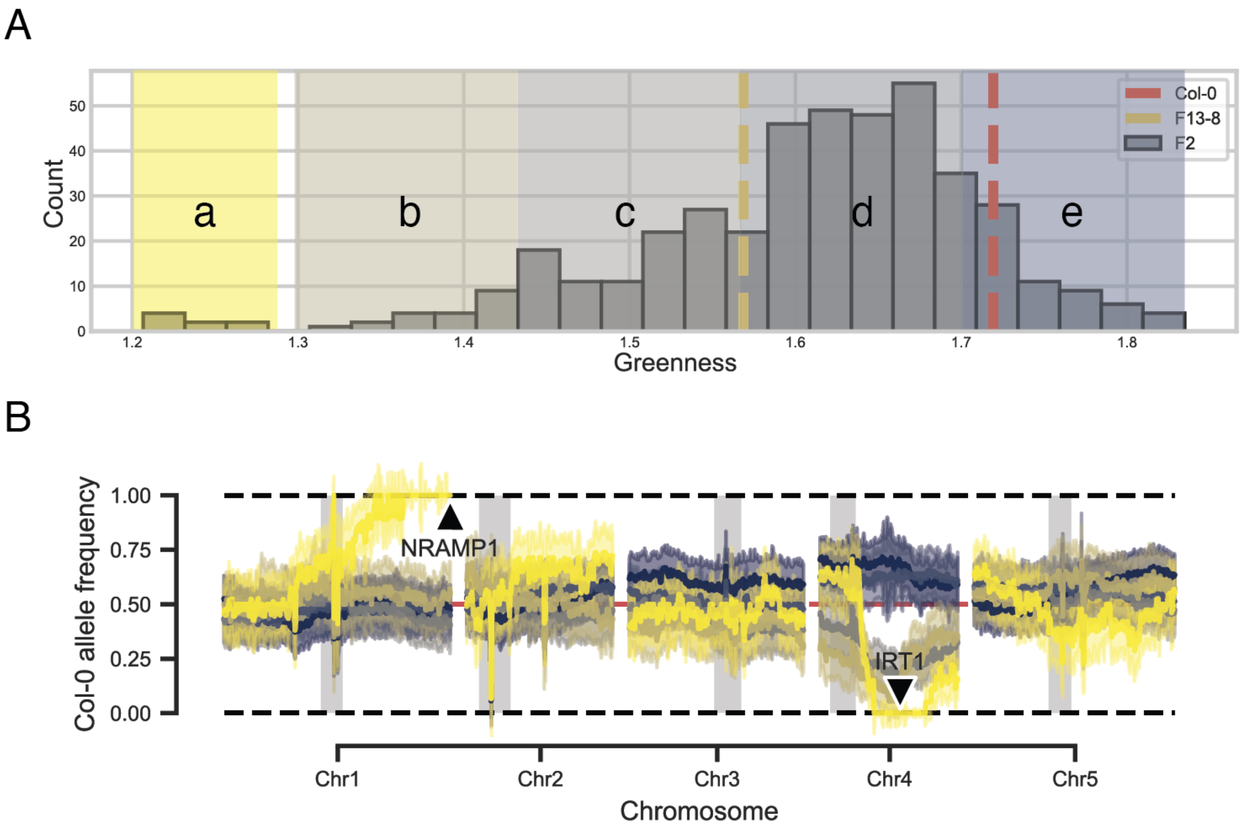

Fig. S7. BSA in the F2 population derived from the cross between Col-0 and F13-8.

(**A**) Variation in greenness in 454 F2 progenies grown in standard potting mix and greenhouse conditions (16 hours of light, 21 ºC). The red and the yellow dashed lines represent the average of the greenness values for Col-0 and F13-8 respectively. The pictures were taken 24 days after sowing. The different pools are color coded and annotated from a to e. (**B**) Visualization of the Col-0 allele frequency in the different pools. Each pool is color coded according to the previous panel. The line represents the median Col-0 allele frequency using a 200 kb sliding window with a step-size of 5 kb, and the shaded area the corresponding standard deviation. The labelled arrows correspond to *IRT1* and *NRAMP1*. The red dashed line indicates 0.5 allele frequency. Grey rectangles specify the centromeric regions.

A

```
Col-0          1 MASNSALLMKTIFLVLIFVSFAISPATSTAPEECGSESANPCVNKAKALPLKVIAIFVIL
Santo Antão    1 MASNSALLMKTIFLVLIFVSFAISPATSTAPEECGSESANPCVNKAKALPLKVIAIFVIL
Fogo           1 MASNSALLMKTIFLVLIFVSFAISPATSTAPEECGSESANPCVNKAKALPLKVIAIFVIL

Col-0         61 IASMIGVGAPLFSRNVSFLQPDGNIFTIIKCFASGIILGTGFMHVLPDSFEMLSSICLEE
Santo Antão   61 IASMIGVGAPLFSRNVSFLQPDGNIFTIIKCFASGIILGTGFMHVLPDSFEMLSSICLEE
Fogo          61 IASMIGVGAPLFSRNVSFLQPDGNIFTIIKCFASGIILGTGFMHVLPDSFEMLSSICLEE

Col-0        121 NPWHKFPFSGFLAMLSGLITLAIDSMATSLYTSKNAVGIMPHGHGHGHGPANDVTLPIKE
Santo Antão  121 NPWHKFPFSGFLAMLSGLITLAIDSMATSLYTSKNAVGIMPHGHGHGHGPANDVTLPIKD
Fogo         121 NPWHKFPFS---------------------------------------------------

Col-0        181 DDSSNAQLLRYRVIAMVLELGIIVHSVVIGLSLGATSDTCTIKGLIAALCFHQMFEGMGL
Santo Antão  181 DDSSNAQLLRYRVIAMVLELGIIVHSVVIGLSLGATSDTCTIKGLIAALCFHQMFEGMGL
Fogo             ------------------------------------------------------------

Col-0        241 GGCILQAEYTNMKKFVMAFFFAVTTPFGIALGIALSTVYQDNSPKALITVGLLNACSAGL
Santo Antão  241 GGCILQAEYTNMKKFVMAFFFAVTTPFGIALGIALSTVYQDNSPKALITVGLLNACSAGL
Fogo             ------------------------------------------------------------

Col-0        301 LIYMALVDLLAAEFMGPKLQGSIKMQFKCLIAALLGCGGMSIIAKWA
Santo Antão  301 LIYMALVDLLAAEFMGPKLQGSIKMQFKCLIAALLGCGGMSIIAKWA
Fogo             -----------------------------------------------
```

B

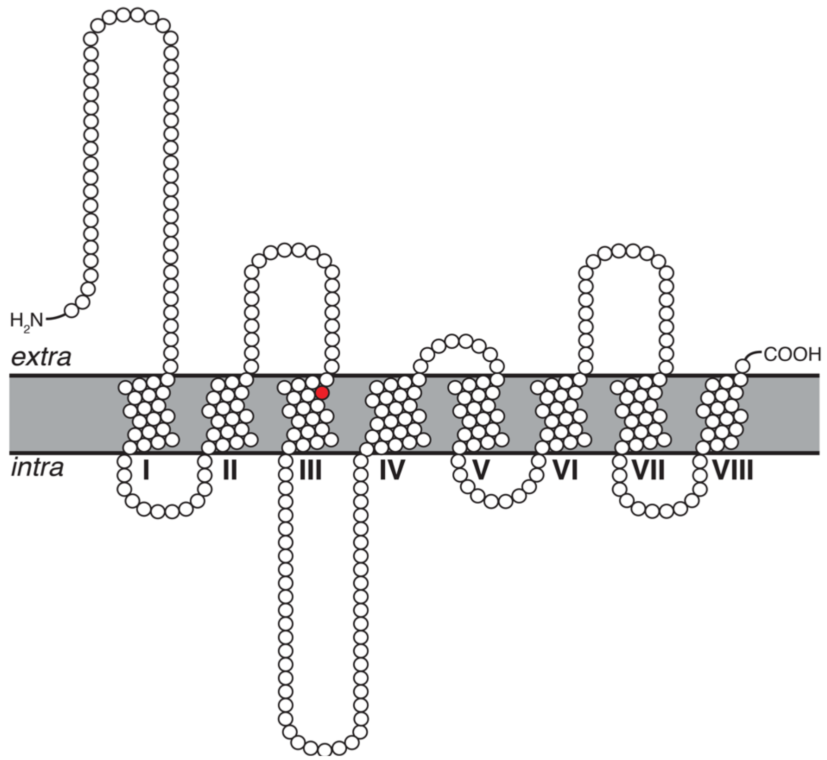

Fig. S8. Functional variant identified on chromosome 4 in F13-8.

(**A**) Multiple alignments of IRT1 protein sequences from Fogo and Santo Antão plants compared to Col-0. (**B**) Schematic diagram of the IRT1 protein with the premature stop codon (G130X) highlighted in red.

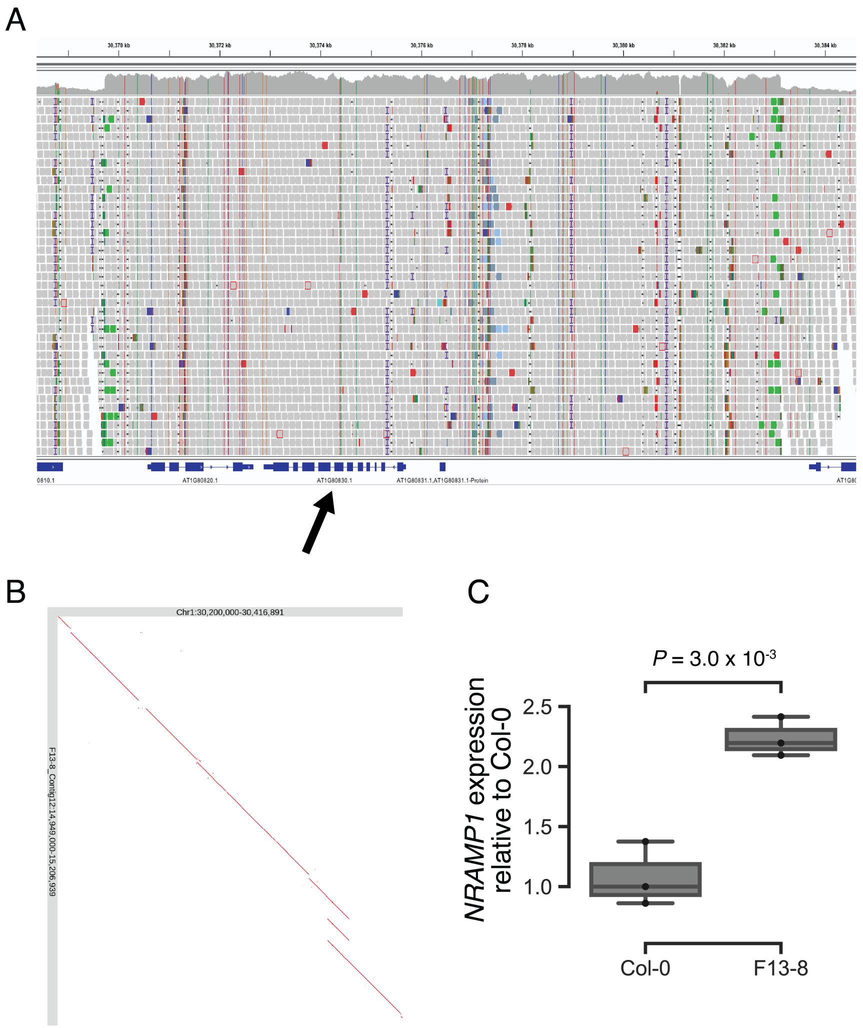

Fig. S9. Functional variant identified on chromosome 1 in F13-8.

(**A**) IGV browser view of F13-8 short-read-sequenced genome showing the tandem duplication located at *NRAMP1*. The arrow indicates *NRAMP1*. The reads are aligned to the TAIR10 reference genome. (**B**) Dot-plots at the end of chromosome 1 in F13-8 (TAIR10:30 200 000-30 416 891). The x and y axes correspond to the position at chromosome 1 for the TAIR10 reference and the *de novo* assembled genome of F13-8, respectively. (**C**) Difference in *NRAMP1* expression in the roots of 12 days old seedlings grown on MS media (3 biological replicates, 3 technical replicates per sample). *NRAMP1* mRNA levels were normalized to the *PP2A* gene and compared to Col-0. Statistical significance was conducted with a Student's *t*-test (p-value indicated).

```
Col-0           1  MAATGSGRSQFISSSGGNRSFSNSPLIENSDSNQIIVSEKKSWKNFFAYLGPGFLVSIAY
Santo Antão     1  MAATGSGRSQFISSSGGNRSFSNSPLIENSDSNQIIVSEKKSWKNFFAYLGPGFLVSIAY
Fogo            1  MAATGSGRSQFISSSGGNRSFSNSPLIENSDSNQIIVSEKKSWKNFFAYLGPGFLVSIAY

Col-0          61  IDPGNFETDLQAGAHYKYELLWIILVASCAALVIQSLAANLGVVTGKHLAEQCRAEYSKV
Santo Antão    61  IDPGNFETDLQAGAHYKYELLWIILVASCAALVIQSLAANLGVVTGKHLAEQCRAEYSKV
Fogo           61  IDPGNFETDLQAGAHYKYELLWIILVASCAALVIQSLAANLGVVTGKHLAEQCRAEYSKV

Col-0         121  PNFMLWVVAEIAVVACDIPEVIGTAFALNMLFSIPVWIGVLLTGLSTLILLALQKYGVRK
Santo Antão   121  PNFMLWVVAEIAVVACDIPEVIGTAFALNMLFSIPVWIGVLLTGLSTLILLALQKYGVRK
Fogo          121  PNFMLWVVAEIAVVACDIPEVIGTAFALNMLFSIPVWIGVLLTGLSTLILLALQKYGVRK

Col-0         181  LEFLIAFLVFTIAICFFVELHYSKPDPGEVLHGLFVPQLKGNGATGLAISLLGAMVMPHN
Santo Antão   181  LEFLIAFLVFTIAICFFVEIHYSKPDPGEVLHGLFVPQLKGNGATGLAISLLGAMVMPHN
Fogo          181  LEFLIAFLVFTIAICFFVEIHYSKPDPGEVLHGLFVPQLKGNGATGLAISLLGAMVMPHN

Col-0         241  LFLHSALVLSRKIPRSASGIKEACRFYLIESGLALMVAFLINVSVISVSGAVCNAPNLSP
Santo Antão   241  LFLHSALVLSRKIPRSASGIKEACRFYLIESGLALMVAFLINVSVISVSGAVCNAPNLSP
Fogo          241  LFLHSALVLSRKIPRSASGIKEACRFYLIESGLALMVAFLINVSVISVSGAVCNAPNLSP

Col-0         301  EDRANCEDLDLNKASFLLRNVVGKWSSKLFAIALLASGQSSTITGTYAGQYVMQGFLDLR
Santo Antão   301  EDRANCEDLDLNKASFLLRNVVGKWSSKLFAIALLASGQSSTITGTYAGQYVMQGFLDLR
Fogo          301  EDRANCEDLDLNKASFLLRNVVGKWSSKLFAIALLASGQSSTITGTYAGQYVMQGFLDLR

Col-0         361  LEPWLRNLLTRCLAIIPSLIVALIGGSAGAGKLIIIASMILSFELPFALVPLLKFTSCKT
Santo Antão   361  LEPWLRNLLTRCLAIIPSLIVALIGGSAGAGKLIIIASMILSFELPFALVPLLKFTSCKT
Fogo          361  LEPWLRNLLTRCLAIIPSLIVALIGGSAGAGKLIIIASMILSFELPFALVPLLKFTSCKT

Col-0         421  KMGSHVNPMAITALTWVIGGLIMGINIYYLVSSFIKLLIHSHMKLILVVFCGILGFAGIA
Santo Antão   421  KMGSHVNPMAITALTWVIGGLIMGINIYYLVSSFIKLLIHSHMKLILVVFCGILGFAGIA
Fogo          421  KMGSHVNPMAITALTWVIGGLIMGINIYYLVSSFIKLLIHSHMKLILVVFCGILGFAGIA

Col-0         481  LYLAAIAYLVFRKNRVATSLLISRDSQNVETLPRQDIVNMQLPCRVSTSDVD
Santo Antão   481  LYLAAIAYLVFRKNRVATSLLISRDSQNVETLPRQDIVNMQLPCRVSTSDVD
Fogo          481  LYLAAIAYLVFRKNRVATSLLISRDSQNVETLPRQDIVNMQLPCRVSTSDVD
```

Fig. S10. NRAMP1 protein sequences are identical within Cape Verdean accessions.
Multiple alignments of NRAMP1 protein sequences from Fogo and Santo Antão plants compared to Col-0. Predicted transmembrane domains are highlighted in red.

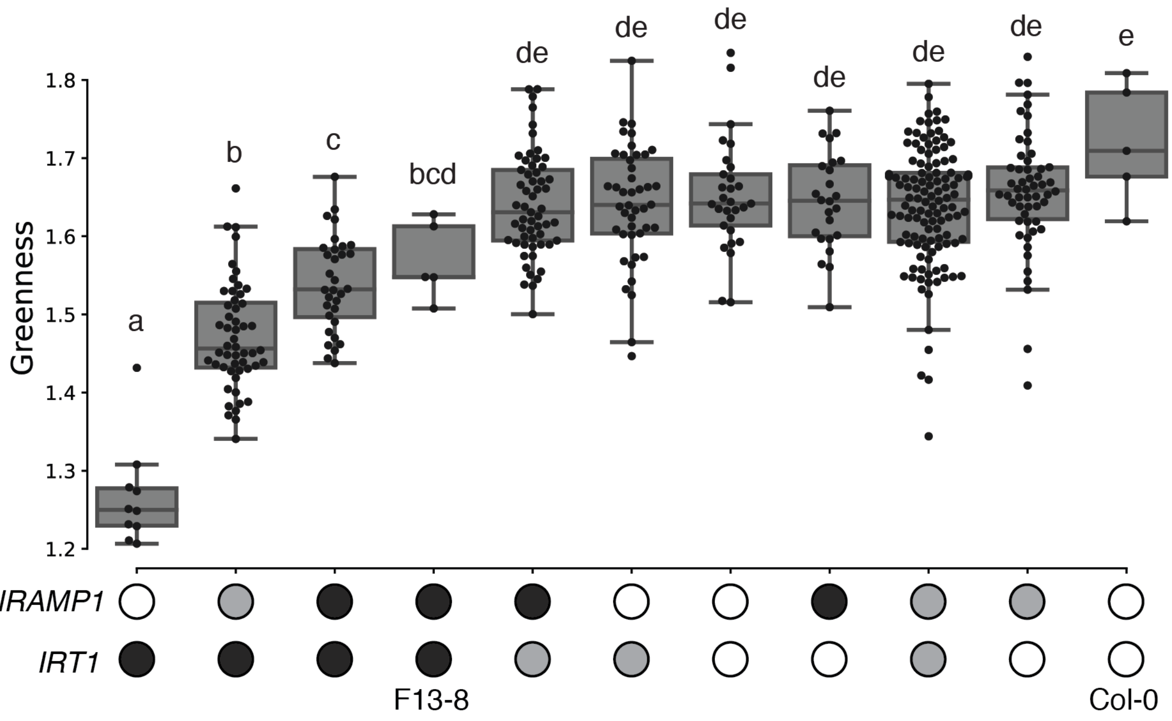

Fig. S11. Chlorosis grouped by the different combinations at *IRT1-NRAMP1* in the F2 progeny derived from the cross between F13-8 and Col-0.

Greenness was evaluated by quantitative image analysis (Green/Red). F2 individuals grown in standard potting mix and greenhouse conditions (16 hours of light, 21 ºC). F2 individuals were genotyped at *NRAMP1* and *IRT1* by dCAPS. Black circles correspond to individuals homozygous for the F13-8 allele, white circles correspond to individuals homozygous for the Col-0 allele and grey circles correspond to heterozygous individuals. The parents F13-8 and Col-0 are indicated, the rest corresponds to F2 individuals. Five replicates of each of the F2 parents (F13-8 and Col-0) have been included in the analysis for comparison. Statistical significance was conducted with a two-way ANOVA followed by a Tukey-adjusted multiple-comparison. Groups sharing a letter are not significantly different. Significance level used 5%.

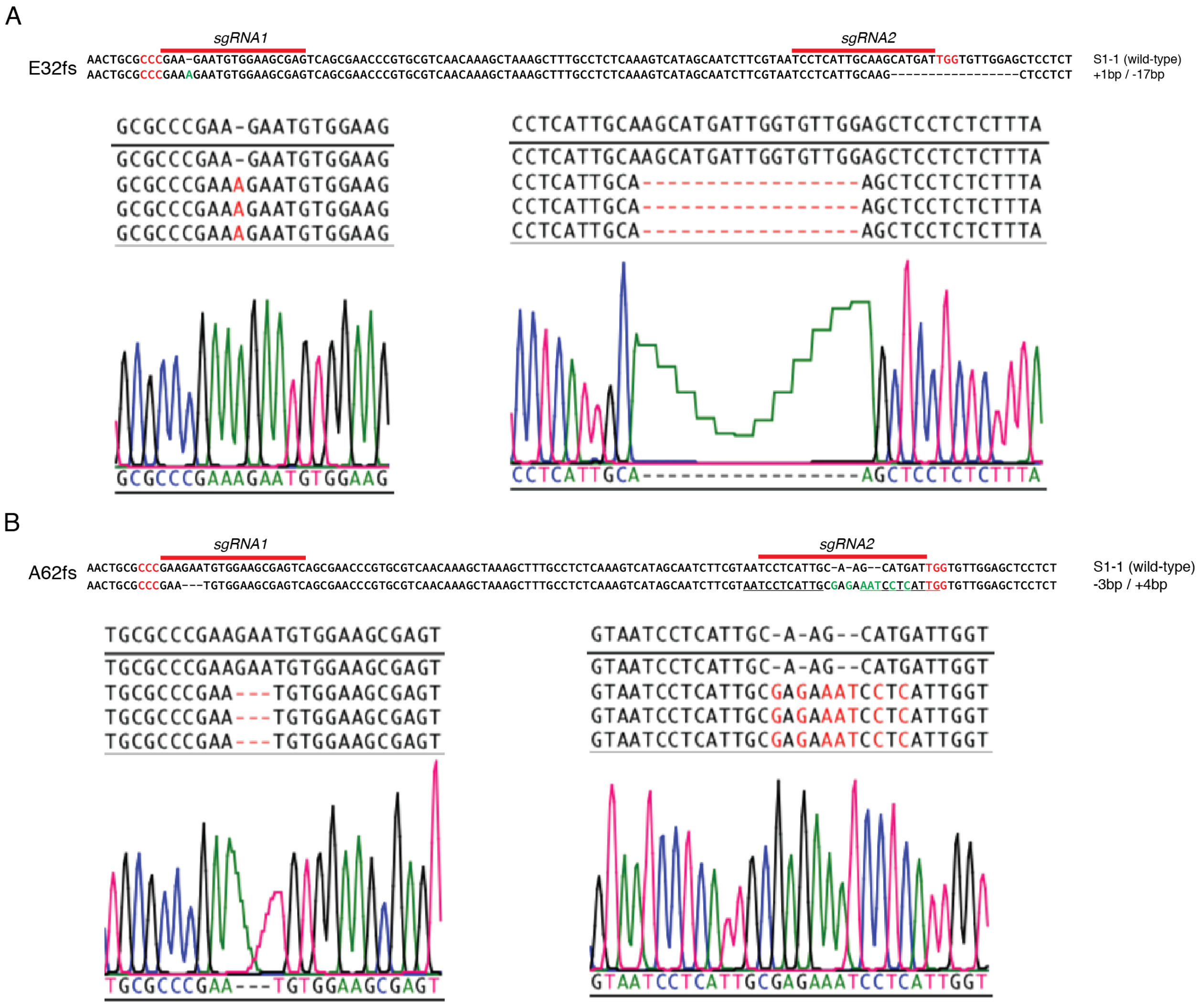

Fig. S12. *IRT1* null alleles generated with CRISPR-Cas9.

(**A**) and (**B**) CRISPR-Cas9 induced mutations at *IRT1* in S1-1 (Santo Antão). The top panels represent the *IRT1* alleles generated, *IRT1* E32fs in (A) and *IRT1* A62fs in (B). The protospacer adjacent motifs (PAM) are highlighted in red and the inserted nucleotides in green. The genomic regions targeted by the *sgRNA1* and *sgRNA2* are annotated in red. The bottom panels correspond to Sanger-sequencing results.

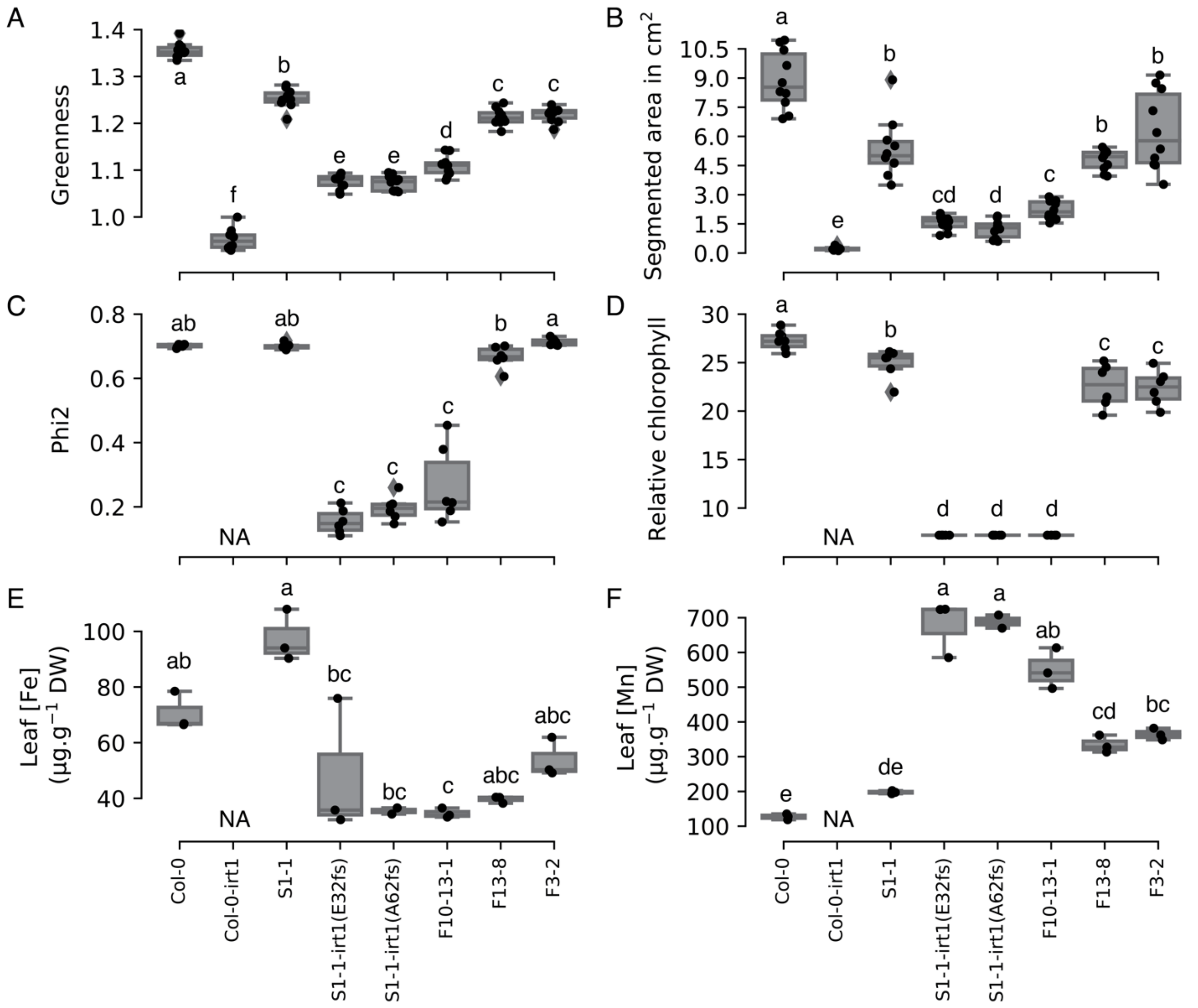

Fig. S13. S1-1-*irt1* chlorosis characterization on standard potting mix.

Variation in (**A**) greenness, (**B**) segmented area in cm^2, (**C**) quantum yield of photosystem II (Phi2) and (**D**) relative chlorophyll content in Col-0, Col-0-*irt1*, S1-1, S1-1-*irt1*(E32fs), S1-1-*irt1*(A62fs), F10-13-1, F13-8 and F3-2 38 days after sowing. Note that the Col-0-*irt1* mutant was too small for multispeQ and ionomic analyzes, and relative chlorophyll content measurements reached detection limit for S1-1-*irt1*(E32fs) and S1-1-*irt1*(A62fs). Variation in Fe (**E**) and Mn (**F**) in the leaves in $\mu g \bullet g^{-1}$ of dry weight (ppm). In E and F, each dot represents a pool of 2 leaves from 8 plants. The statistical significance was conducted with a Kruskal-Wallis test. Significance level used 5% after Bonferroni correction. Groups sharing a letter are not significantly different.

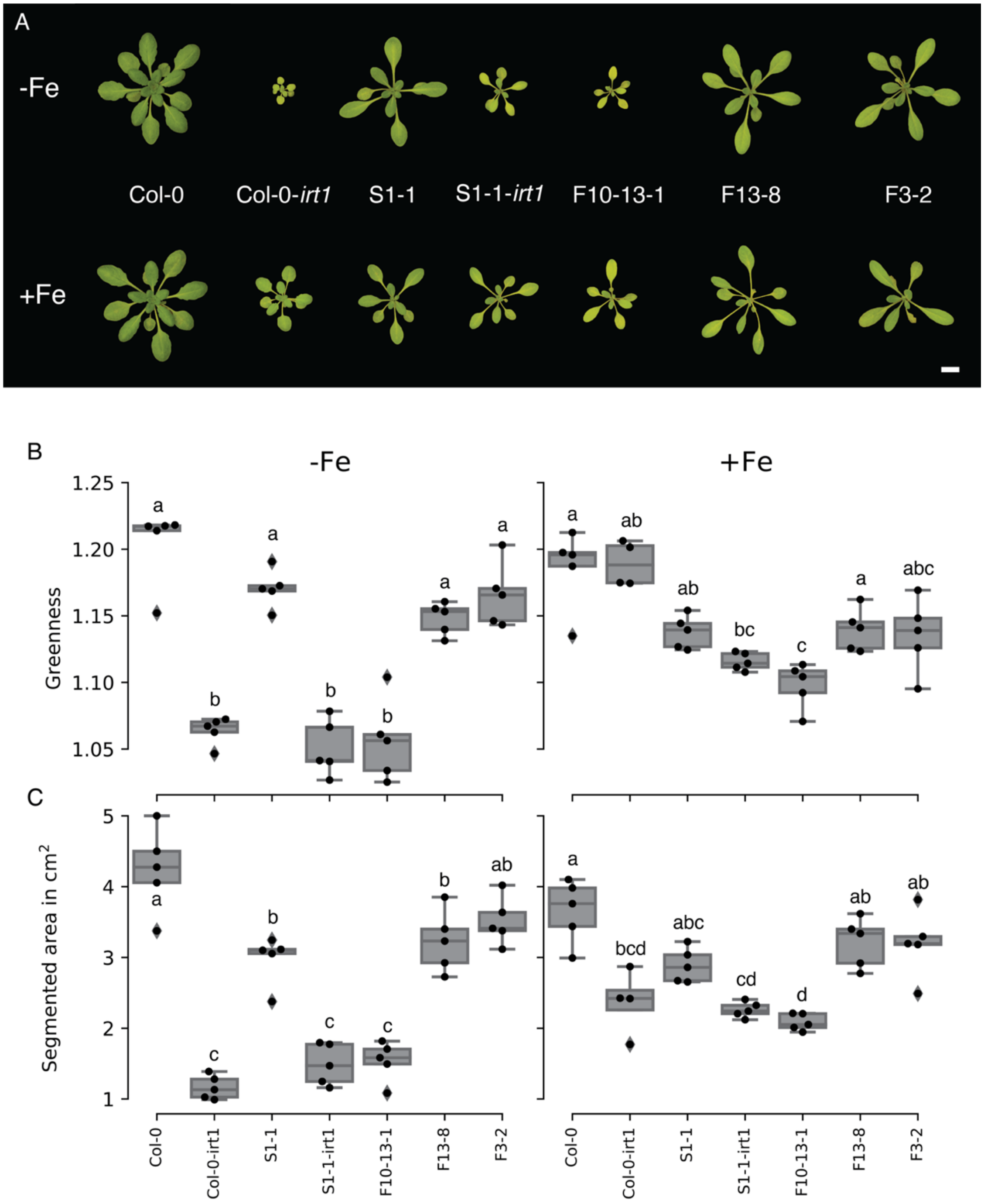

Fig. S14. Supplementation of Fe partially reduces chlorosis in Fogo plants.

(**A**) Pictures of Col-0, Col-0-*irt1*, S1-1, S1-1-*irt1*, F10-13-1, F13-8 and F3-2 seedlings grown on standard potting mix 36 days after sowing. Scale bar = 1 cm. Variation in (**B**) greenness and segmented area in cm^2 (**C**) between Col-0, Col-0-*irt1*, S1-1, S1-1-*irt1*, F10-13-1, F13-8 and F3-2. Plants were supplemented with Fe (+Fe) or not (-Fe). The statistical significance was conducted with a Kruskal-Wallis test. Significance level used 5% after Bonferroni correction. Groups sharing a letter are not significantly different.

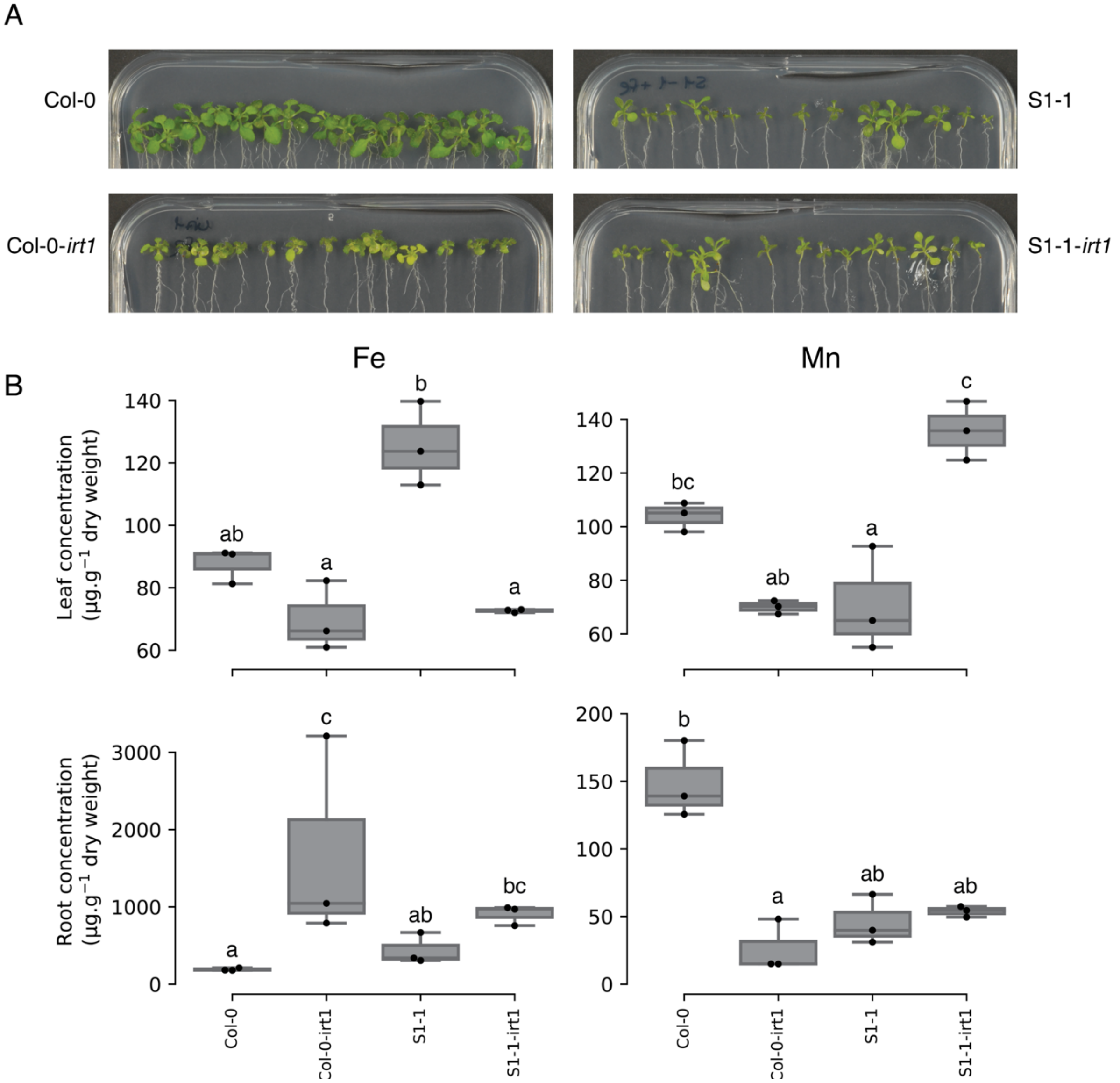

Fig. S15. Impact of loss of function of *IRT1* on Fe and Mn concentrations in leaves and roots of seedlings grown on modified Hoagland media.

(**A**) Pictures of Col-0, Col-0-*irt1*, S1-1 and S1-1-*irt1* seedlings grown on agar-solidified Hoagland media 17 days after sowing. (**B**) Variation in Fe and Mn in the leaves and the roots in µg•g^{-1} of dry weight (ppm). The statistical significance was conducted with a Kruskal-Wallis test. Significance level used 5% after Bonferroni correction. Groups sharing a letter are not significantly different.

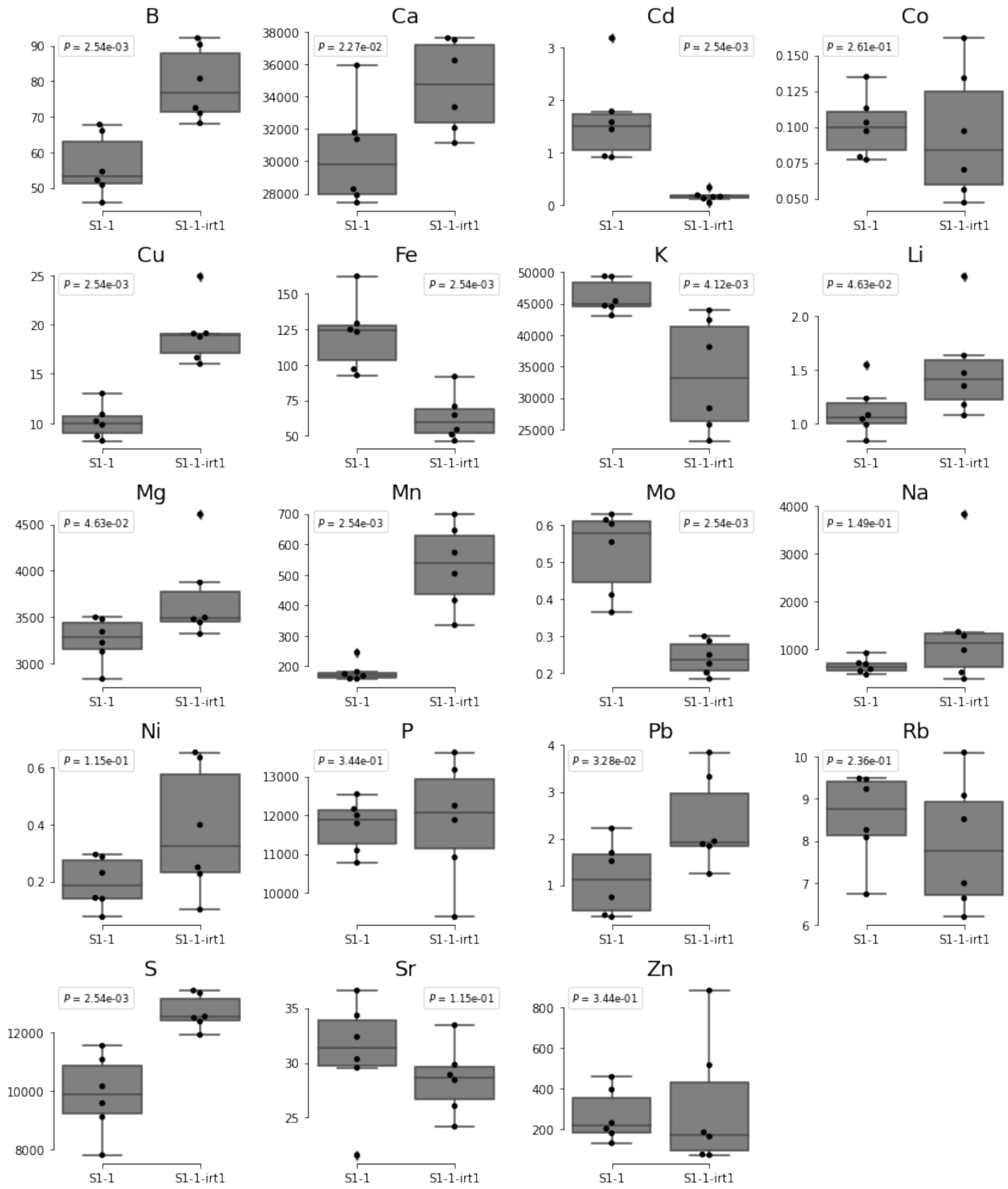

Fig. S16. Impact of loss of IRT1 on the leaf ionome of S1-1 grown on standard potting mix. Difference between S1-1 and S1-1-*irt1* for 19 elements in the leaves. Values are shown in μg•g^{-1} of dry weight (ppm). *P* = p-value for Mann-Whitney-Wilcoxon test.

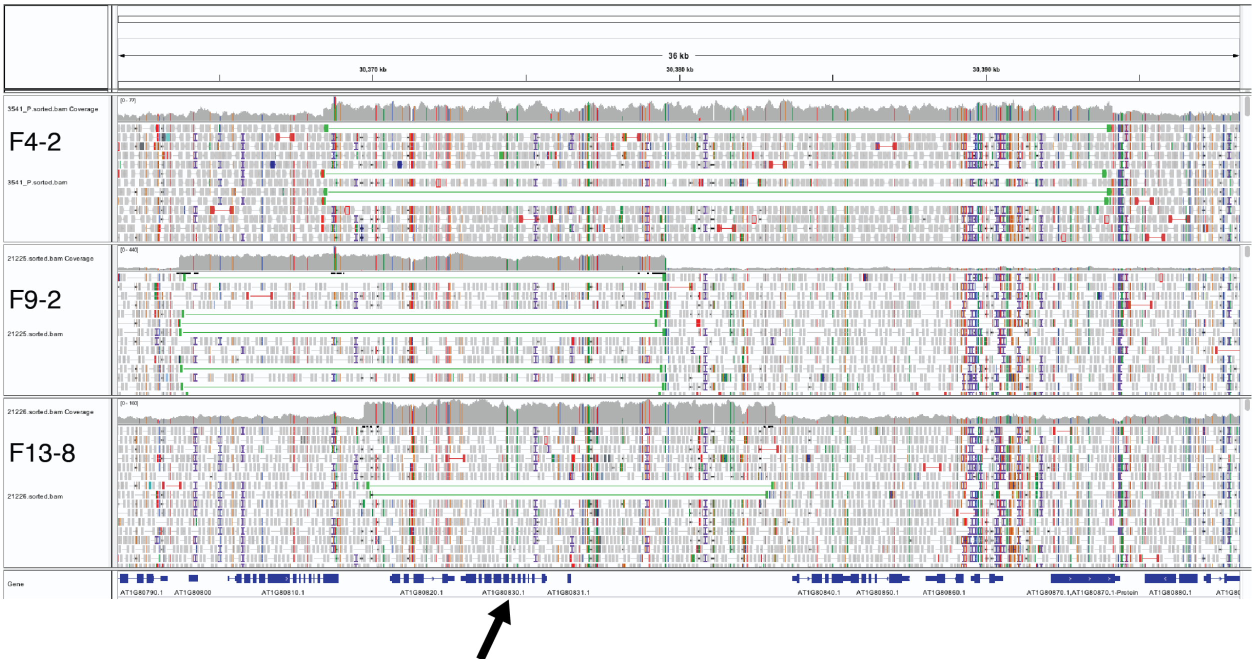

Fig. S17. *NRAMP1* tandem duplication haplotypes in the Fogo population.

IGV browser view of the genomic region at *NRAMP1* in F4-2 (*CT*-TD haplotype - 2 copies), F9-2 (*ATG*-TD haplotype - 5 copies) and F13-8 (*AAGACTAA*-TD haplotype - 3 copies). The arrow indicates *NRAMP1*. The reads are shown as pairs and aligned to the TAIR10 reference genome.

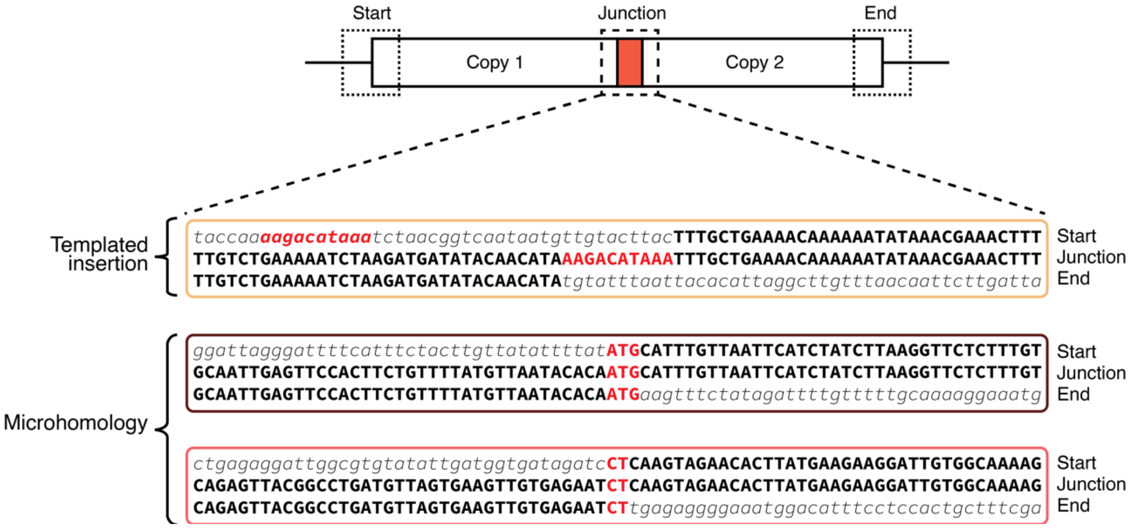

Fig. S18. Breakpoint junctions at the tandem duplications surrounding *NRAMP1*.

Breakpoint junctions (in red) at the *NRAMP1* TD haplotypes discovered in Fogo. *AAGACATAA*-TD contains a 10 bp junction (AAGACATAA) templated 27 bp upstream of the breakpoint while *ATG*-TD and *CT*-TD show two different microhomology-mediated junctions. The breakpoint junctions are aligned to the start and the end of the breakpoints.

A

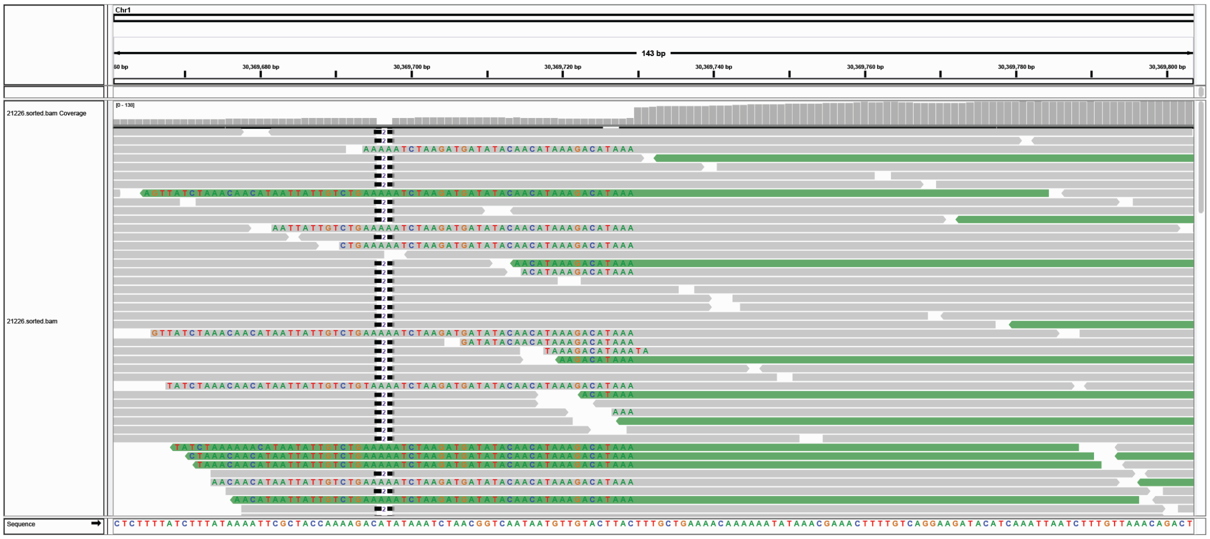

B

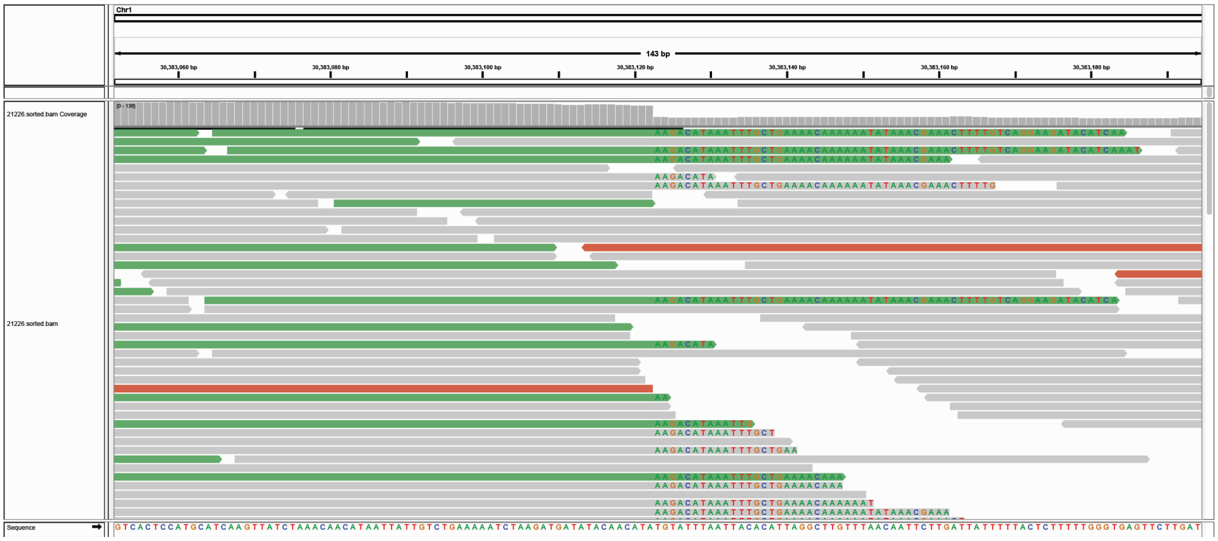

Fig. S19. Breakpoints at the *AAGACTAA*-TD haplotype in F13-8 (3 copies).

IGV browser view of the breakpoints upstream (**A**) and downstream (**B**) of the tandem duplication showing the discordant reads (in green) and the soft-clipped bases at the breakpoints (multicolor).

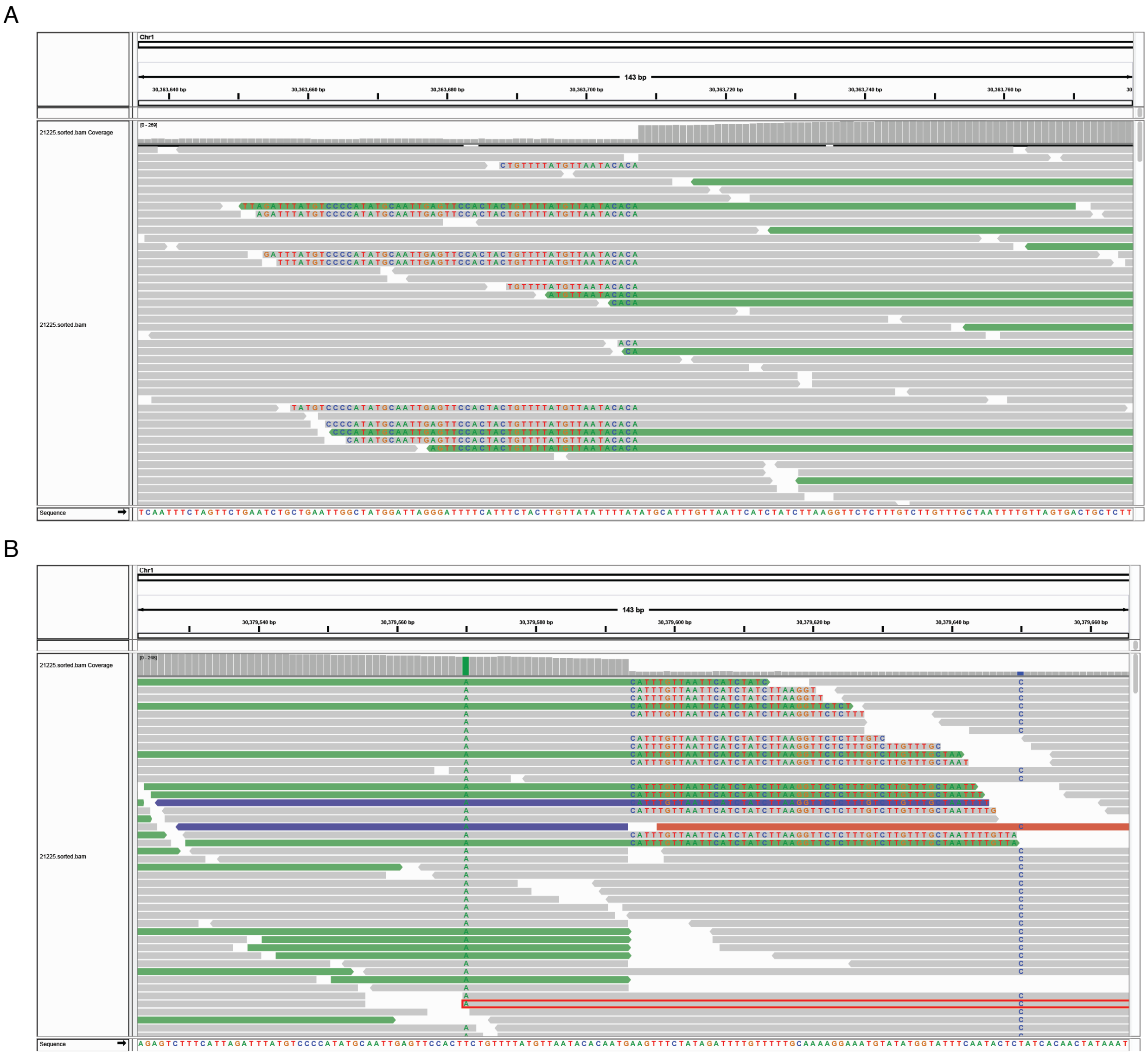

Fig. S20. Breakpoints at the *ATG*-TD haplotype in F9-2 (5 copies).

IGV browser view of the breakpoints upstream (**A**) and downstream (**B**) of the tandem duplication showing the discordant reads (in green) and the soft-clipped bases at the breakpoints (multicolor).

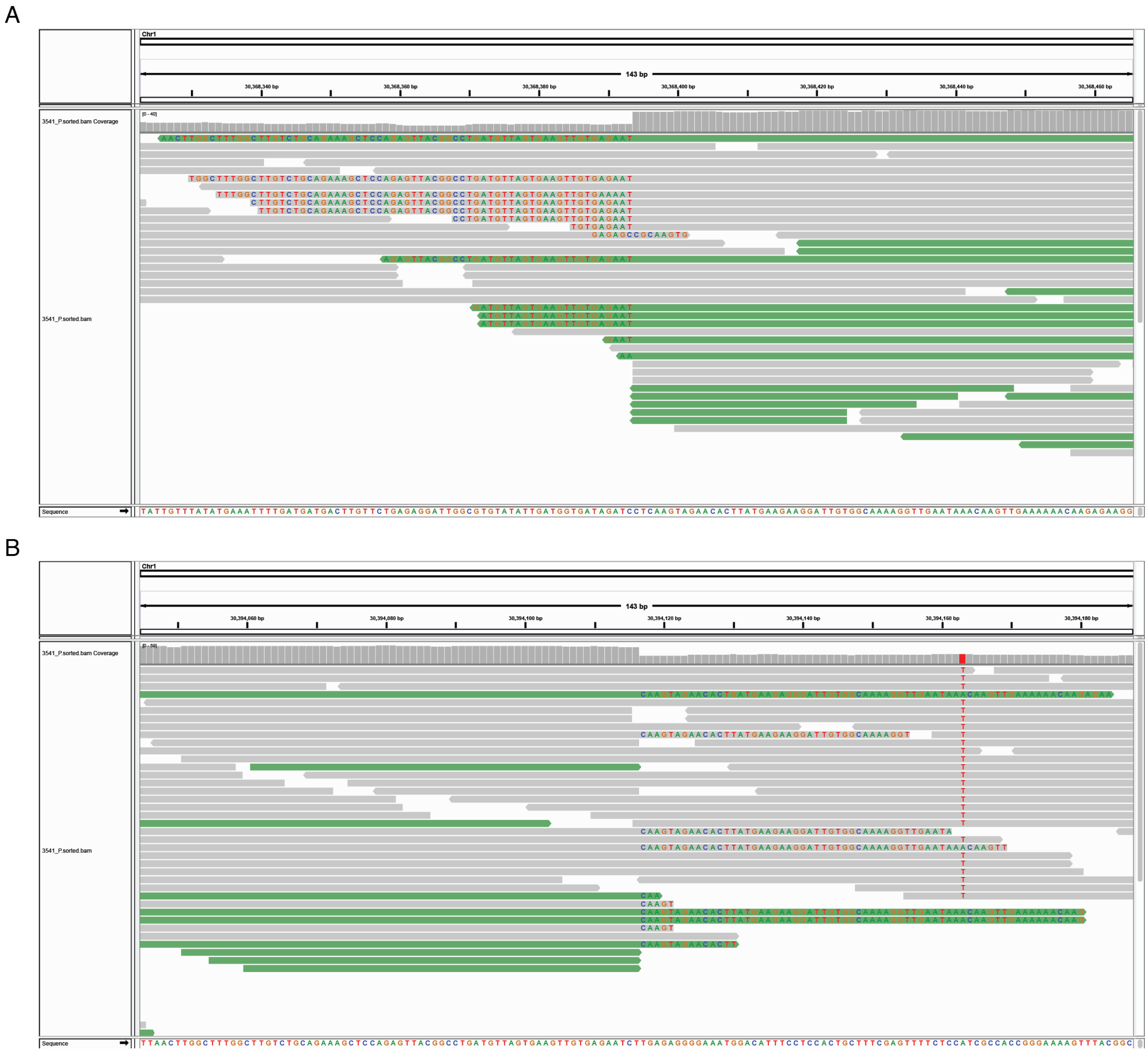

Fig. S21. Breakpoints at the *CT*-TD *NRAMP1* haplotype in F4-2 (2 copies).

IGV browser view of the breakpoints upstream (**A**) and downstream (**B**) of the tandem duplication showing the discordant reads (in green) and the soft-clipped bases at the breakpoints (multicolor).

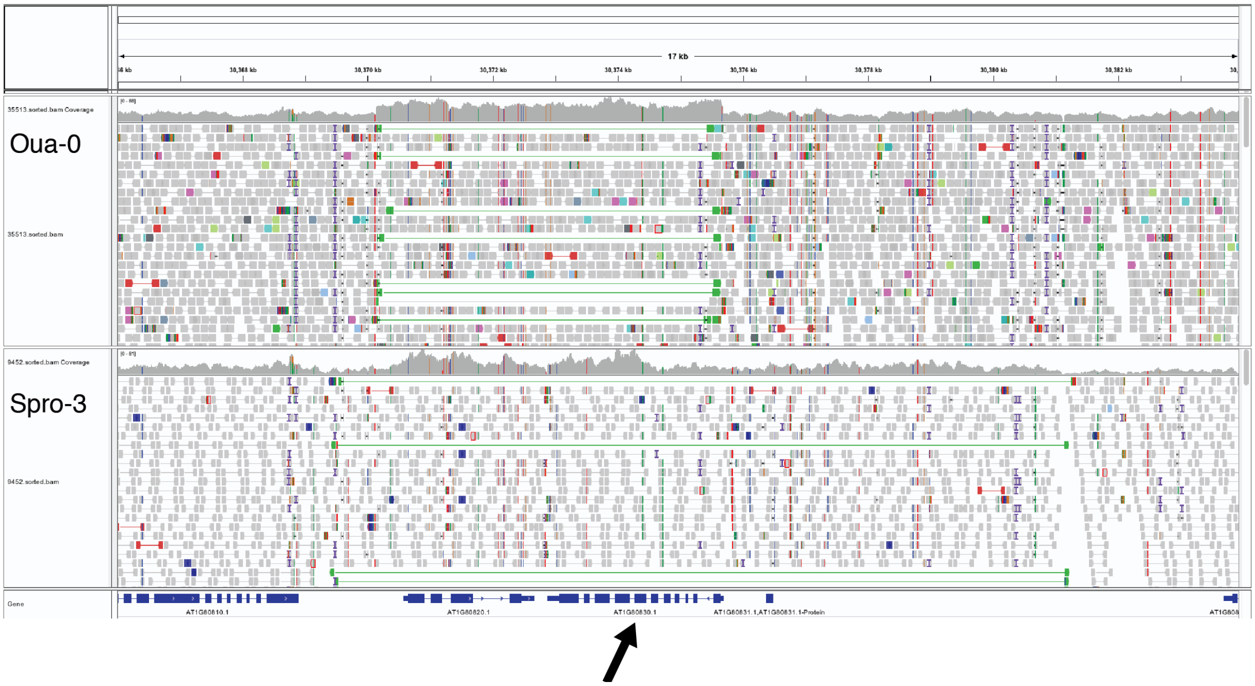

Fig. S22. ***NRAMP1*** **copy number variation outside of Fogo.**

IGV browser view of the genomic region at *NRAMP1* in Oua-0 (35513) and Spro-3 (9452). The reads are shown as pairs and aligned to the TAIR10 reference genome. The arrow indicates *NRAMP1*.

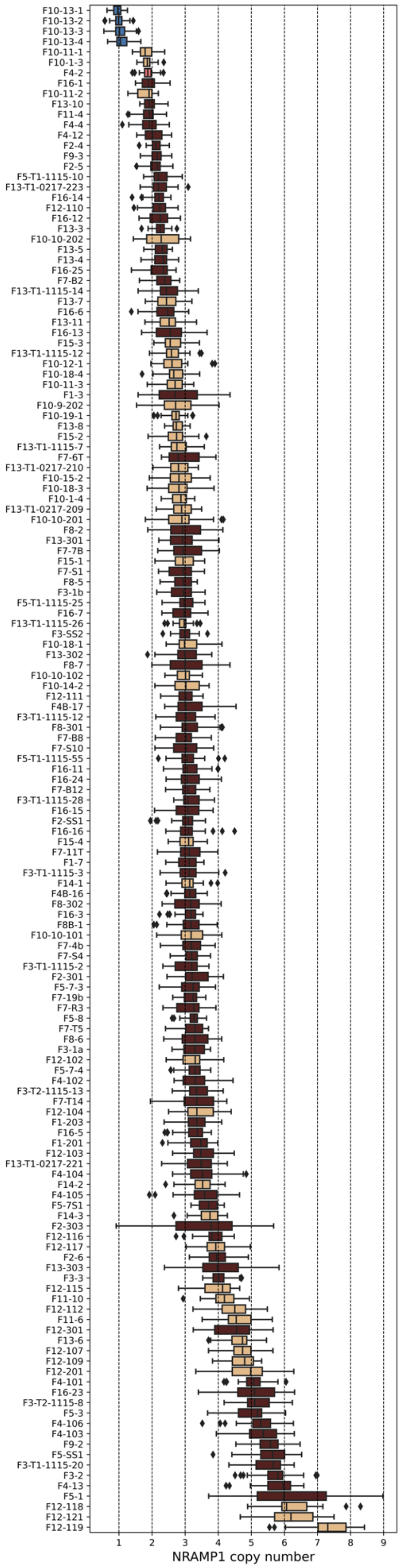

Fig. S23. ***NRAMP1*** **copy number variation in the Fogo population.**

NRAMP1 copy number estimates in the Fogo population based on the analysis described in Fig. 4B. The different *NRAMP1* haplotypes are annotated by color (*AAGACATAA*-TD in beige, *ATG*-TD in burgundy, *CT*-TD in pink and the single *NRAMP1* copy in blue).

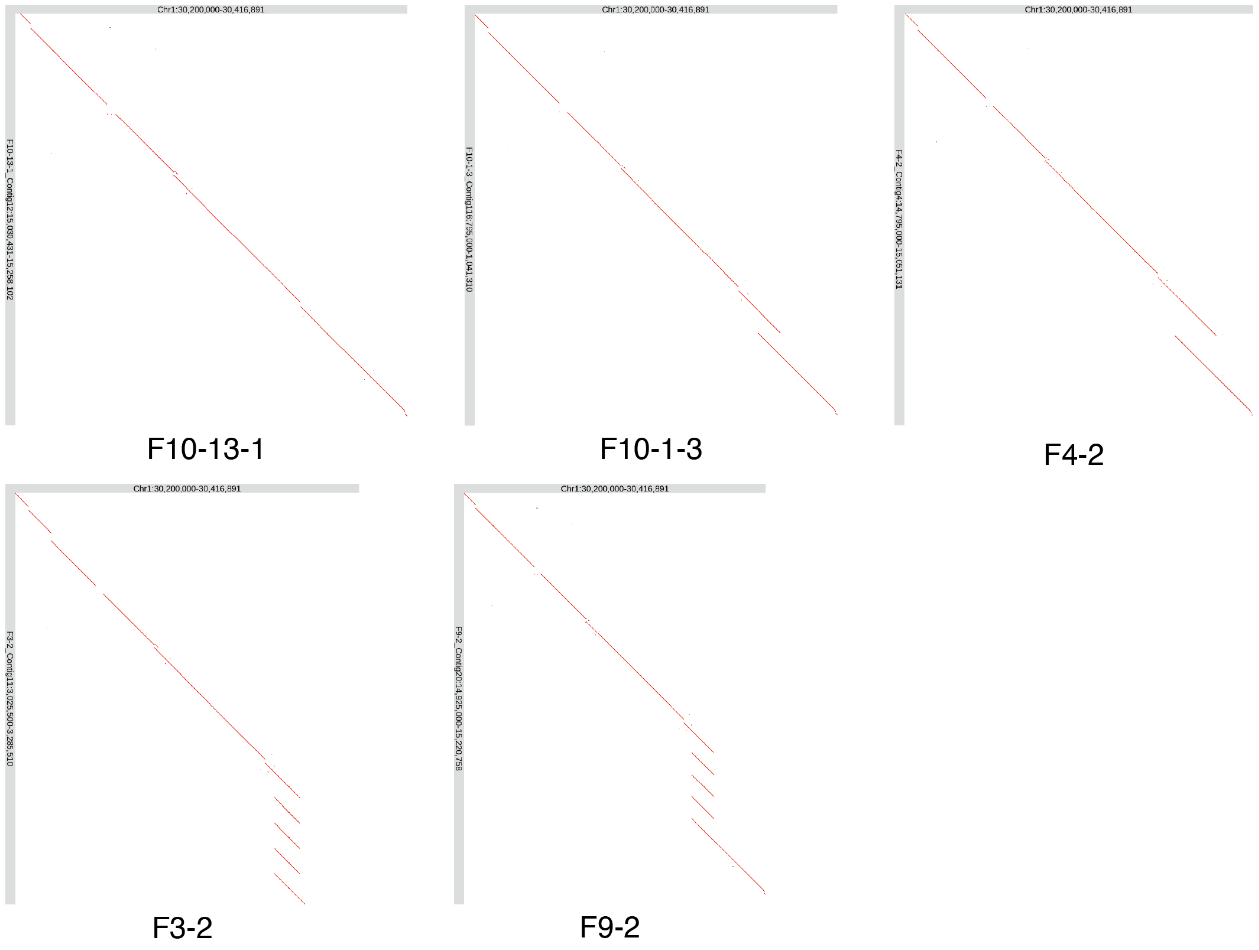

Fig. S24. Genomic region at *NRAMP1* in Nanopore-based *de novo* assemblies.

Dot-plots at the end of chromosome 1 for F10-13-1 (1 *NRAMP1* copy), F10-1-3 (*AAGACATAA*-TD - 2 *NRAMP1* copies), F4-2 (*CT*-TD haplotype - 2 *NRAMP1* copies), F3-2 (*ATG*-TD - 5 *NRAMP1* copies) and F9-2 (*ATG*-TD - 5 *NRAMP1* copies) sequences compared to the TAIR10 reference genome. The x and y axes correspond to the position at chromosome 1 for the TAIR10 reference and the *de novo* assembled genomes, respectively.

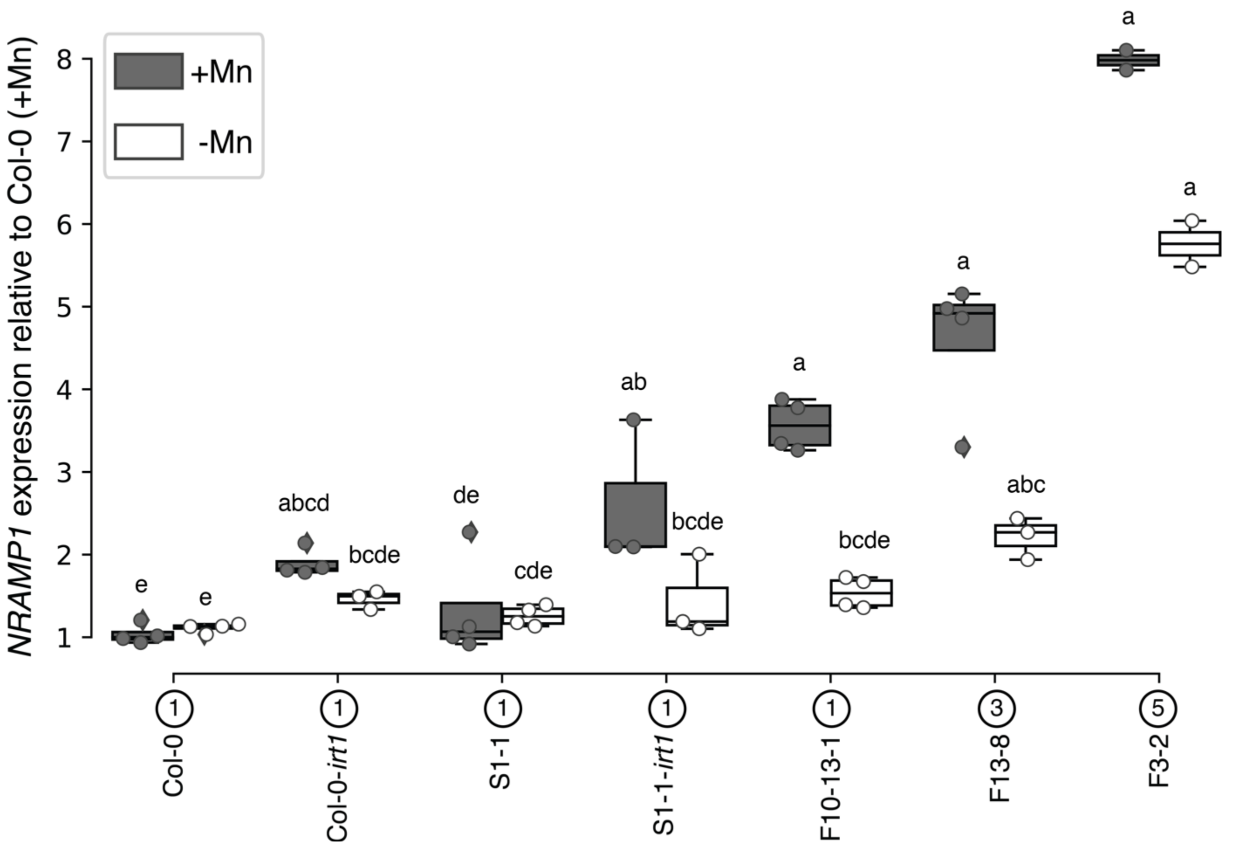

Fig. S25. *NRAMP1* expression analysis.

NRAMP1 expression in roots of 14-day-old seedlings grown on agar-solidified Hoagland media. mRNA levels were quantified with a probe-based digital PCR assay (2 to 4 biological replicates). *NRAMP1* copy numbers are indicated within the circles. *NRAMP1* mRNA levels were normalized to the *PP2A* gene and compared to Col-0 (+Mn). The statistical significance was conducted with a Kruskal-Wallis test. Significance level used 5% after Bonferroni correction. Groups sharing a letter are not significantly different.

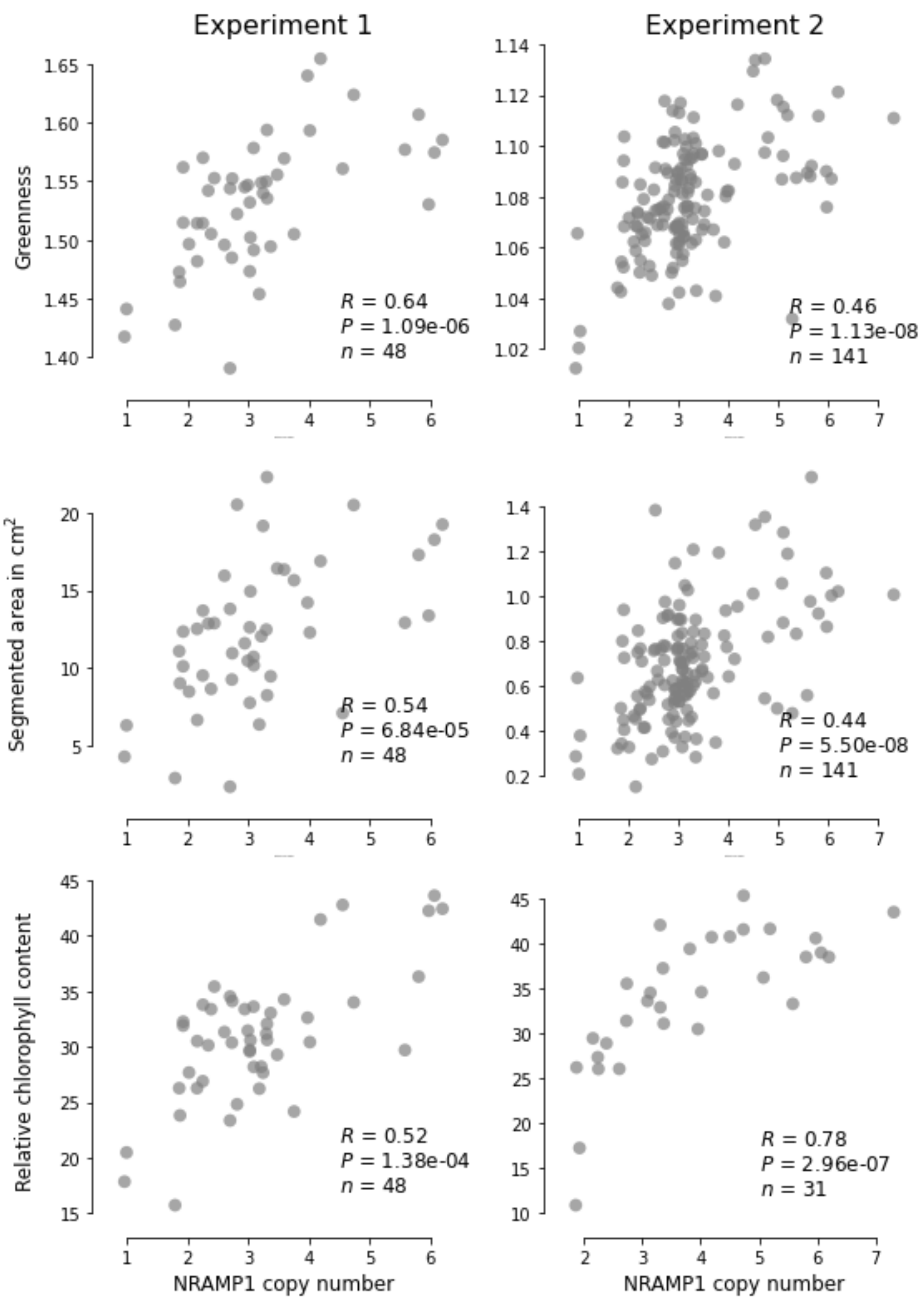

Fig. S26. Correlation between *NRAMP1* copy number and chlorosis variation in the Fogo population.

Correlation between *NRAMP1* copy number and greenness, segmented area in cm^2 and relative chlorophyll content in two independent experiments. *R* = Spearman's rho, *P* = p-value and *n* = number of genotypes.

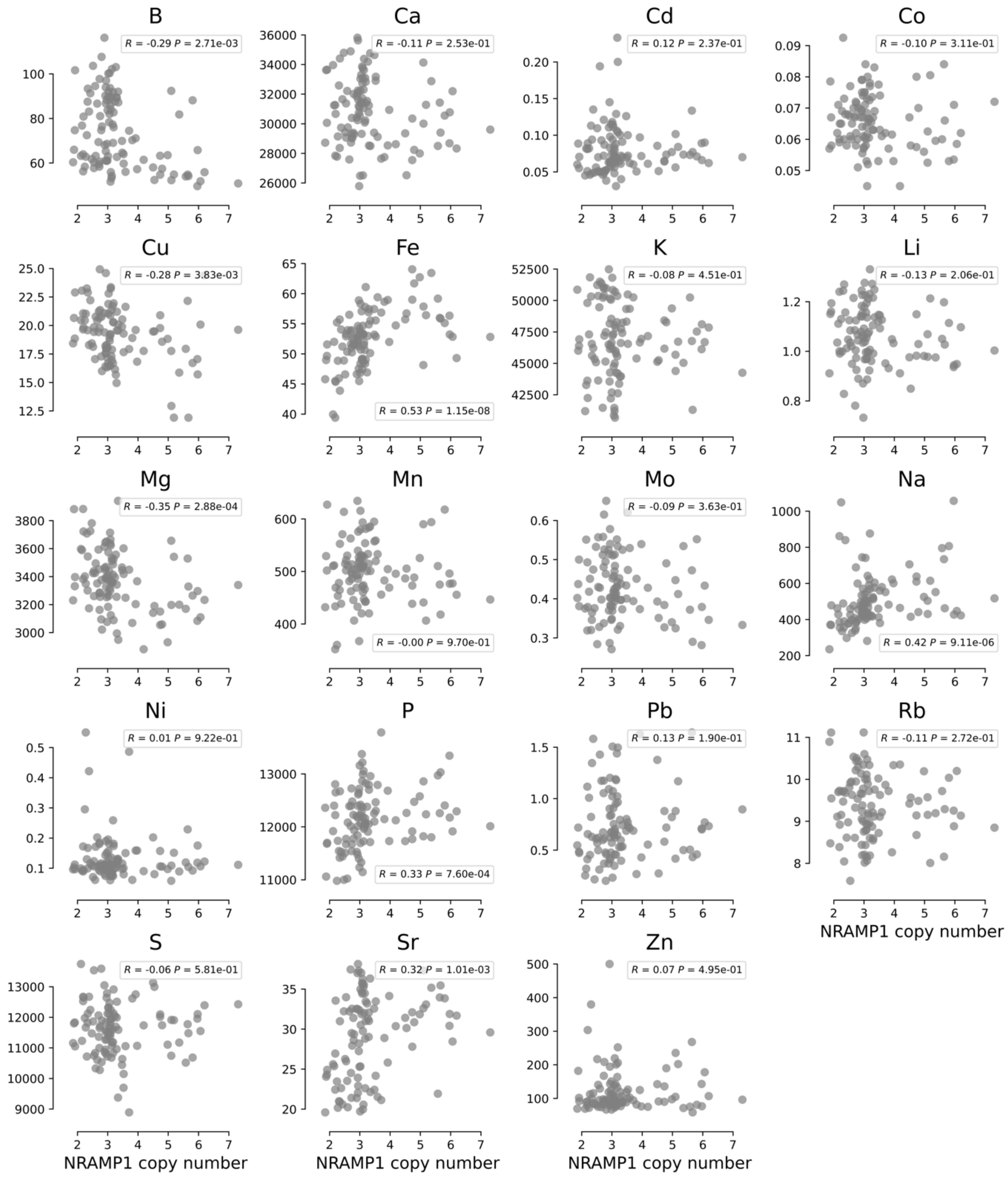

Fig. S27. Correlation between *NRAMP1* copy number and 19 elements in the leaves of Fogo natural accessions.

The plants were grown on standard potting mix in controlled growth chamber conditions (12 hours of light, 21 ºC at day, 14 ºC at night, 70% humidity). The leaf elements concentrations on the y axis are indicated in $\mu g \bullet g^{-1}$ of dry weight (ppm) and correspond to the median across replicates per accession. The values on the x axis correspond to the *NRAMP1* copy numbers. The tissue was harvested five to six weeks after sowing. *R* = Spearman's rho, *P* = p-value, *n* =103).

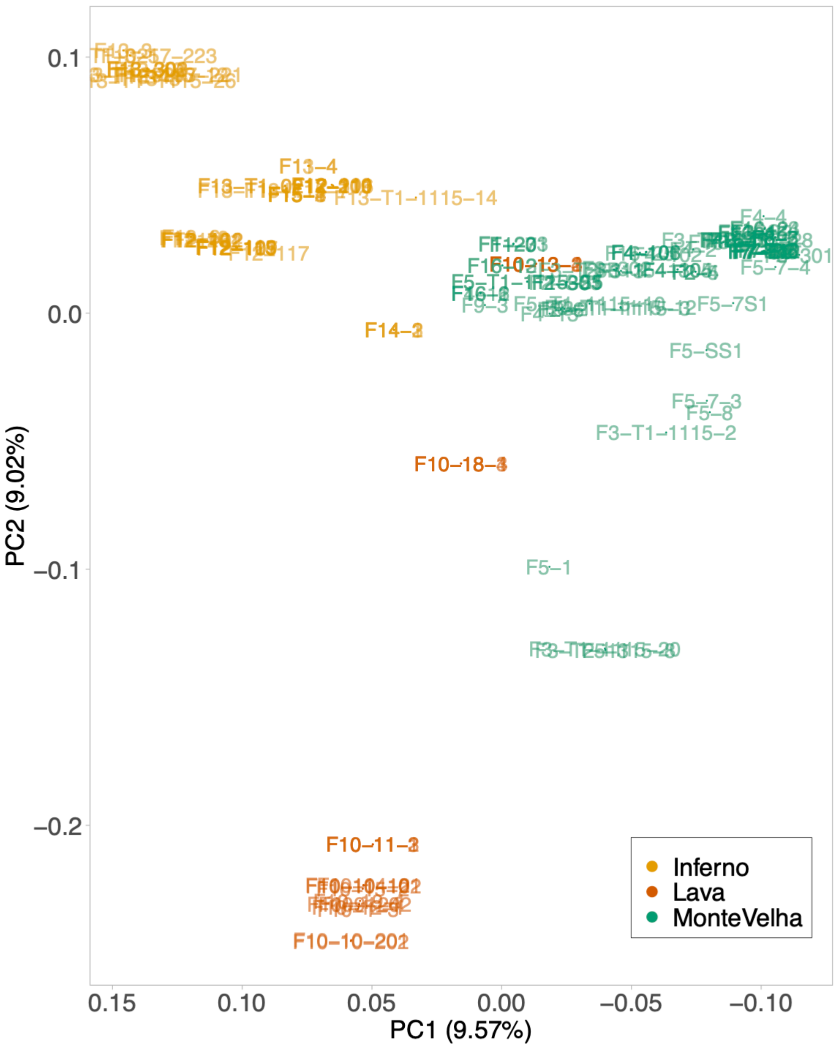

Fig. S28. First two principal components of PCA based on LD-pruned genome-wide SNPs. PCA as in Fig. 5B with the name of the accessions indicated.

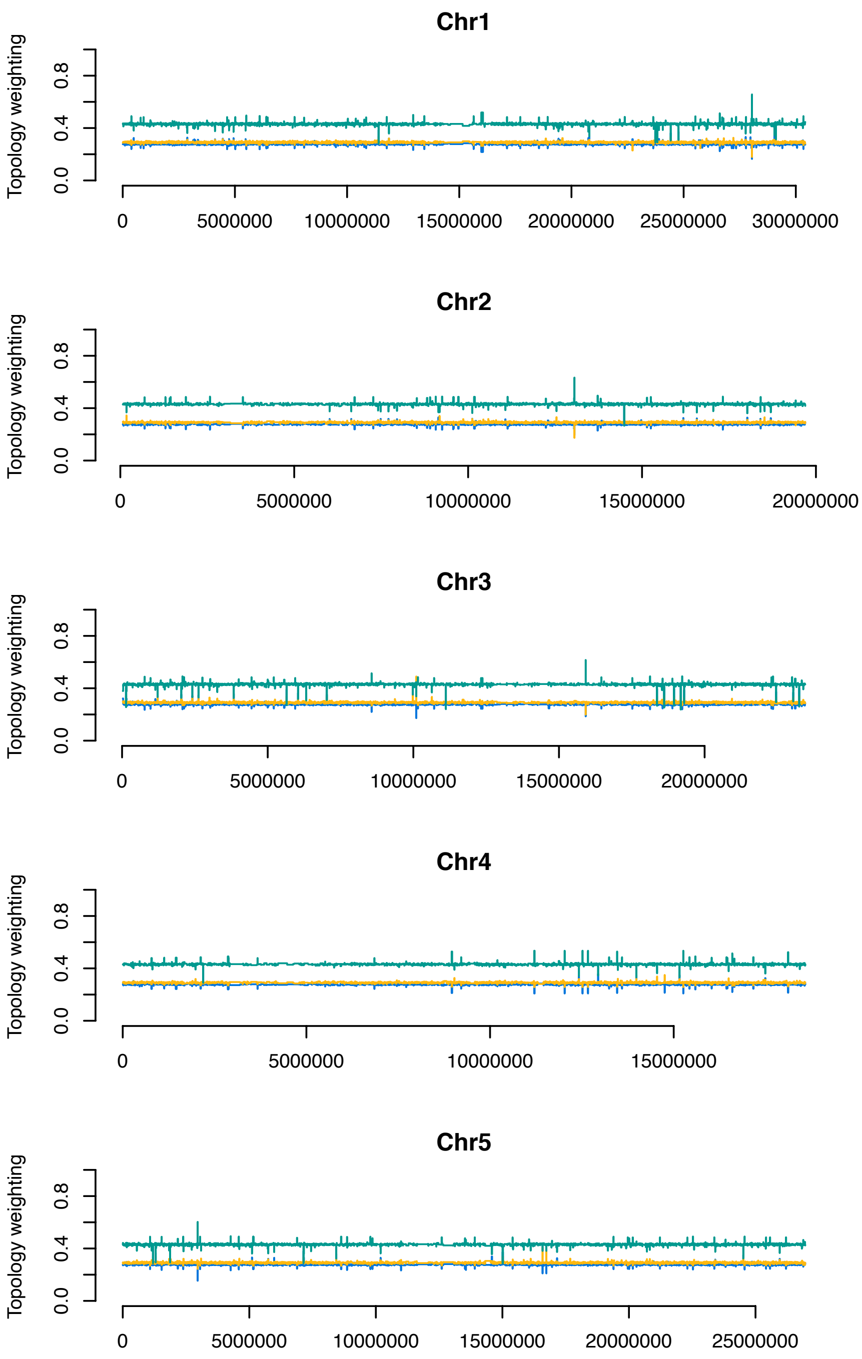

Fig. S29. Twisst genome-wide sliding window analysis of weightings of all topologies. Colored lines indicate three distinct topologies: Topology1 (green), Topology2 (yellow), and Topology3 (blue).

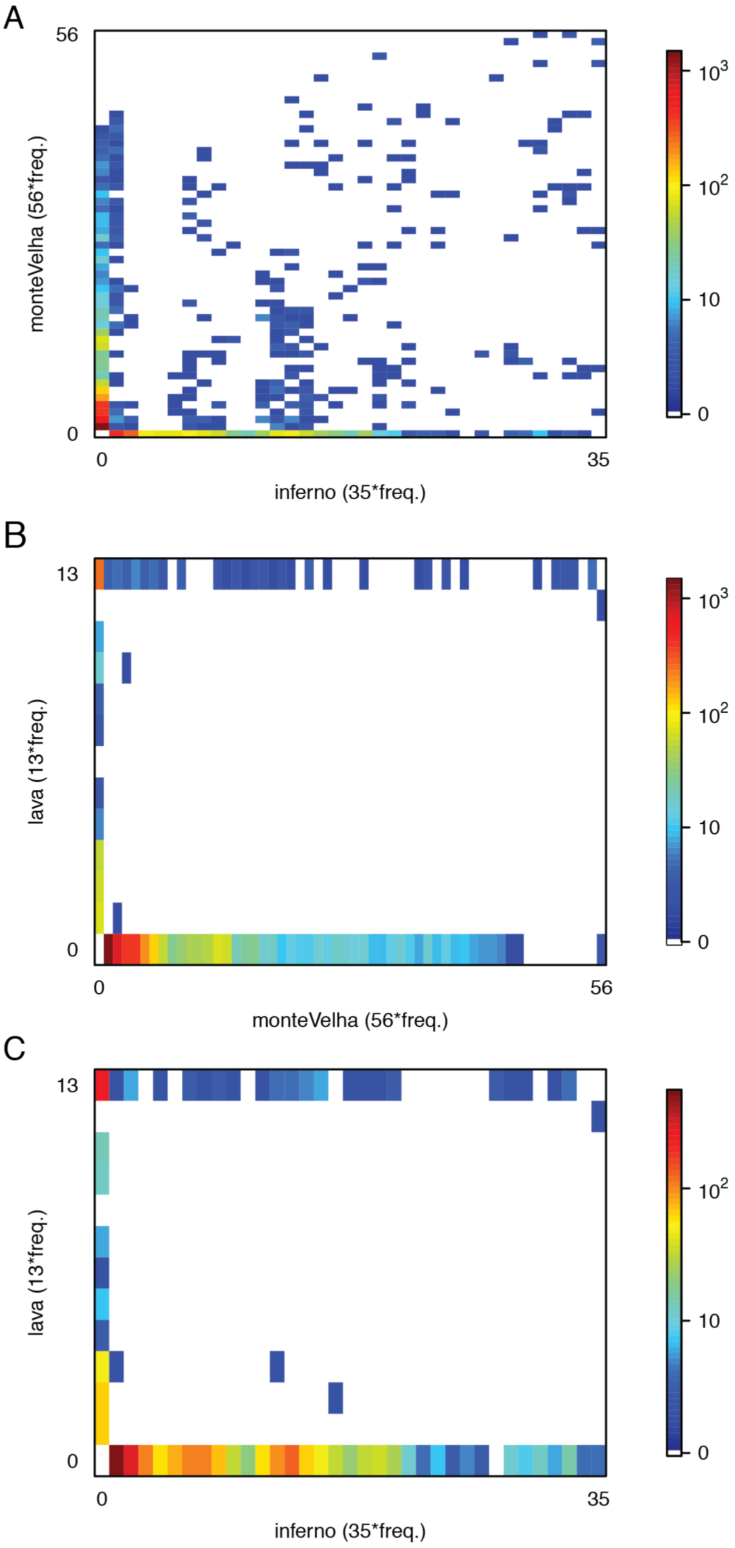

Fig. S30. Joint site frequency spectra between the three Fogo sub-populations.

Joint site frequency spectra between (**A**) Monte Velha and Inferno, (**B**) Monte Velha and Lava and (**C**) Lava and Inferno. The color scale reflects the number of variants.

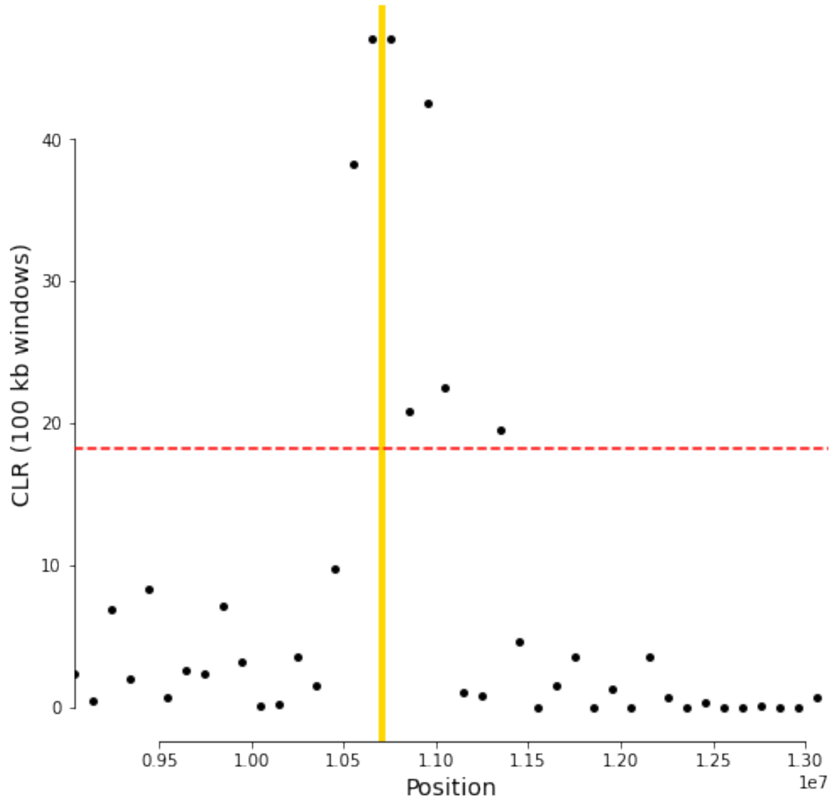

Fig. S31. Zoom at the strongest genome wide signal CLR overlapping with *IRT1*.
CLR with non-overlapping windows of 100 kb. The red dashed line corresponds to the 1% tail of genome-wide CLR. The yellow line indicates the position of *IRT1*. Each dot corresponds to a 100 kb window.

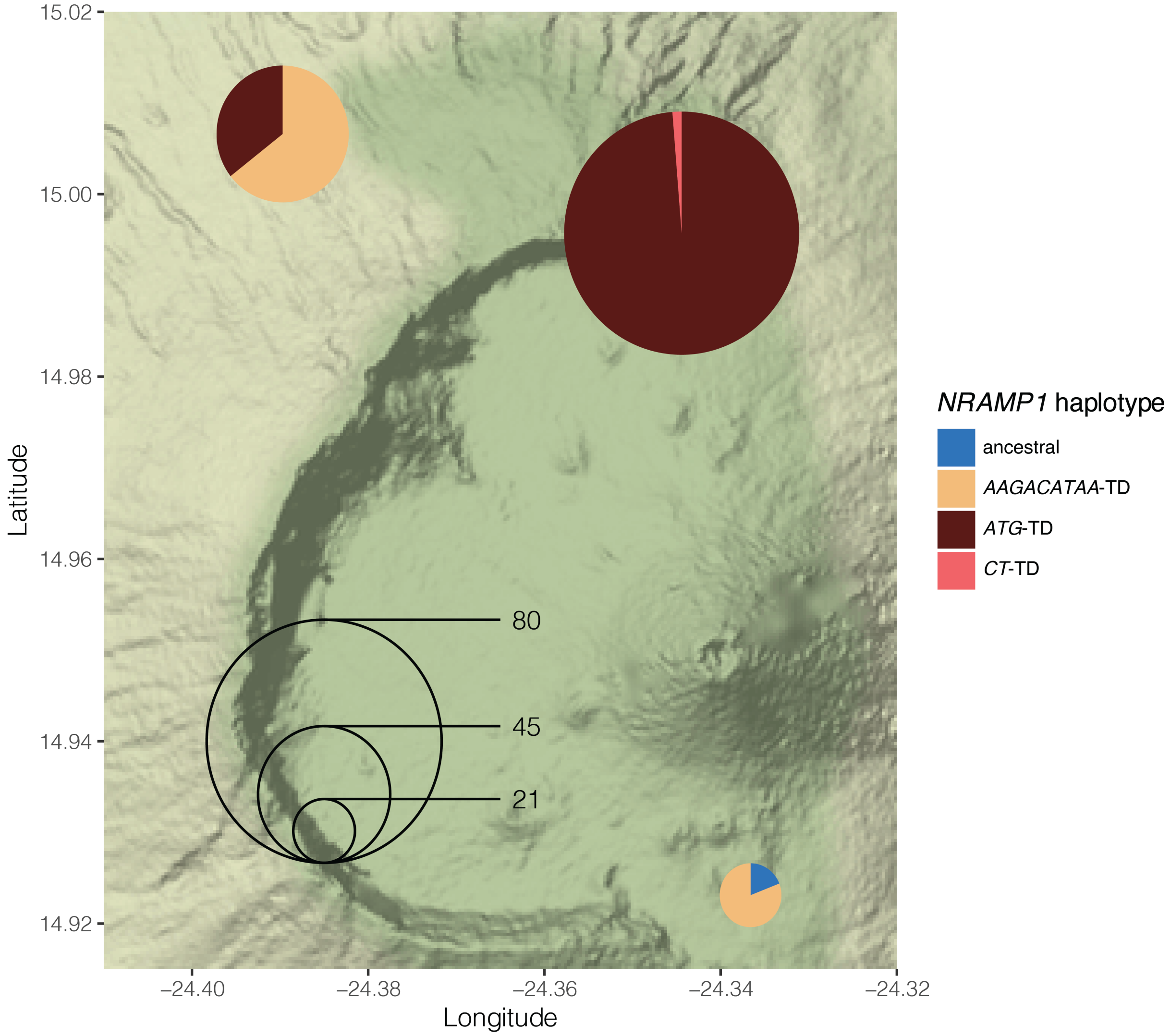

Fig. S32. Geographic distribution of *NRAMP1* TD haplotypes across Fogo.
NRAMP1 TD haplotypes are geographically structured across major geographic regions. Circle's size defines the number of individuals.

Table S1: Contribution of the different elements to the first four principal components.
Table S2: Summary experiment on standard potting mix; SEM=standard error of the mean; P=p-value with Kruskal-Wallis test.
Table S3: pH measurements in topsoil collected in Fogo and Santo Antão.
Table S4: ICP-MS results for 23 water extractable elements in Fogo and Santo Antão soils.
Table S5: Variants at the peak on the chromosome 1 from the bulk segregant analysis.
Table S6: Variants at the peak on the chromosome 4 from the bulk segregant analysis.
Table S7: Structural variants on the chromosome 1 in F13-8 (21226).
Table S8: ANOVA table to test the effect of *IRT1* and *NRAMP1* on chlorosis.
(Df) degree of freedom, (Sum Sq) sum of squares, (Mean Sq) mean of squares.
Table S9: Multiple linear regression to test the epistatic interaction between *IRT1* and *NRAMP1* on chlorosis. H: Heterozygous, F: Homozygous for the Fogo allele.
Table S10: Variation at *IRT1* in Arabidopsis.
Table S11: *NRAMP1* copy number estimations by digital PCR.
Table S12: *NRAMP1* haplotypes in Fogo.
Table S13: Multiple linear regression to test the effect of *NRAMP1* copy number variation on *NRAMP1* transcript levels.
Table S14: Chip heritability estimated with GEMMA using a linear mixed model; PVE corresponds to the proportion of phenotypic variance explained by genotypes or chip heritability; PVE_{NRAMP1} corresponds to the chip heritability using *NRAMP1* copy number as covariate.
Table S15: Variants at the genomic region from CLR overlapping with *IRT1*.
Table S16: Alleles age estimates based on Relate trees.
Table S17: Inferred selection coefficients for *IRT1* 130X using Clues. logLR corresponds to the log likelihood. s corresponds to the selection coefficient.
Table S18: Inferred selection coefficients for *NRAMP1 AAGACATAA*-TD and *ATG*-TD using Clues. logLR corresponds to the log likelihood. s corresponds to the selection coefficient.
Table S19: Primers used in this study.
